# Simultaneous Consonance in Music Perception and Composition

**DOI:** 10.1037/rev0000169

**Published:** 2019-12-23

**Authors:** Peter M. C. Harrison, Marcus T. Pearce

**Affiliations:** 1 School of Electronic Engineering and Computer Science, Queen Mary University of London; 2 School of Electronic Engineering and Computer Science, Queen Mary University of London, and Center for Music in the Brain, Aarhus University

**Keywords:** composition, consonance, dissonance, music, perception

## Abstract

Simultaneous consonance is a salient perceptual phenomenon corresponding to the perceived pleasantness of simultaneously sounding musical tones. Various competing theories of consonance have been proposed over the centuries, but recently a consensus has developed that simultaneous consonance is primarily driven by harmonicity perception. Here we question this view, substantiating our argument by critically reviewing historic consonance research from a broad variety of disciplines, reanalyzing consonance perception data from 4 previous behavioral studies representing more than 500 participants, and modeling three Western musical corpora representing more than 100,000 compositions. We conclude that simultaneous consonance is a composite phenomenon that derives in large part from three phenomena: interference, periodicity/harmonicity, and cultural familiarity. We formalize this conclusion with a computational model that predicts a musical chord’s simultaneous consonance from these three features, and release this model in an open-source R package, *incon*, alongside 15 other computational models also evaluated in this paper. We hope that this package will facilitate further psychological and musicological research into simultaneous consonance.

Simultaneous consonance is a salient perceptual phenomenon that arises from simultaneously sounding musical tones. Consonant tone combinations tend to be perceived as pleasant, stable, and positively valenced; dissonant combinations tend conversely to be perceived as unpleasant, unstable, and negatively valenced. The opposition between consonance and dissonance underlies much of Western music (e.g., Dahlhaus, 1990; Hindemith, 1945; Parncutt & Hair, 2011; Rameau, 1722; Schoenberg, 1978).^1^

Many psychological explanations for simultaneous consonance have been proposed over the centuries, including amplitude fluctuation (Vassilakis, 2001), masking of neighboring partials (Huron, 2001), cultural familiarity (Johnson-Laird, Kang, & Leong, 2012), vocal similarity (Bowling, Purves, & Gill, 2018), fusion of chord tones (Stumpf, 1890), combination tones (Hindemith, 1945), and spectral evenness (Cook, 2009). Recently, however, a consensus is developing that consonance primarily derives from a chord’s harmonicity (Bidelman & Krishnan, 2009; Bowling & Purves, 2015; Cousineau, McDermott, & Peretz, 2012; Lots & Stone, 2008; McDermott, Lehr, & Oxenham, 2010; Stolzenburg, 2015), with this effect potentially being moderated by musical exposure (McDermott et al., 2010; McDermott, Schultz, Undurraga, & Godoy, 2016).

Here we question whether harmonicity is truly sufficient to explain simultaneous consonance perception. First, we critically review historic consonance research from a broad variety of disciplines, including psychoacoustics, cognitive psychology, animal behavior, computational musicology, and ethnomusicology. Second, we reanalyze consonance perception data from four previous studies representing more than 500 participants (Bowling et al., 2018; Johnson-Laird et al., 2012; Lahdelma & Eerola, 2016; Schwartz, Howe, & Purves, 2003). Third, we model chord prevalences in three large musical corpora representing more than 100,000 compositions (Broze & Shanahan, 2013; Burgoyne, 2011; Viro, 2011). On the basis of these analyses, we estimate the degree to which different psychological mechanisms contribute to consonance perception in Western listeners.

Computational modeling is a critical part of our approach. We review the state of the art in consonance modeling, empirically evaluate 20 of these models, and use these models to test competing theories of consonance. Our work results in two new consonance models: a corpus-based cultural familiarity model, and a composite model of consonance perception that captures interference between partials, harmonicity, and cultural familiarity. We release these new models in an accompanying R package, *incon*, alongside new implementations of 14 other models from the literature (see *Software* for details). In doing so, we hope to facilitate future consonance research in both psychology and empirical musicology.

## Musical Terminology

Western music is traditionally notated as collections of atomic musical elements termed *notes*, which are organized along two dimensions: *pitch* and *time.* In performance, these notes are translated into physical sounds termed *tones*, whose pitch and timing reflect the specifications in the musical score. Pitch is the psychological correlate of a waveform’s oscillation frequency, with slow oscillations sounding “low” and fast oscillations sounding “high.”

Western listeners are particularly sensitive to *pitch intervals*, the perceptual correlate of frequency ratios. Correspondingly, a key principle in Western music is *transposition invariance*, the idea that a musical object (e.g., a melody) retains its perceptual identity when its pitches are all shifted (*transposed*) by the same interval.

A particularly important interval is the *octave*, which approximates a 2:1 frequency ratio.^2^ Western listeners perceive a fundamental equivalence between pitches separated by octaves. Correspondingly, a *pitch class* is defined as an equivalence class of pitches under octave transposition. The *pitch-class interval* between two pitch classes is then defined as the smallest possible ascending interval between two pitches belonging to the respective pitch classes.

In Western music theory, a *chord* may be defined as a collection of notes that are sounded simultaneously as tones. The lowest of these notes is termed the *bass note*. Chords may be termed based on their size: For example, the terms *dyad*, *triad*, and *tetrad* denote chords comprising two, three, and four notes respectively. Chords may also be termed according to the representations of their constituent notes: (a) *Pitch sets* represent notes as absolute pitches; (b) *Pitch-class sets* represent notes as pitch classes; and (c) *Chord types* represent notes as intervals from the bass note.

This paper is about the *simultaneous consonance* of musical chords. A collection of notes is said to be *consonant* if the notes “sound well together,” and conversely *dissonant* if the notes “sound poorly together.” In its broadest definitions, consonance is associated with many different musical concepts, including diatonicism, centricism, stability, tension, similarity, and distance (Parncutt & Hair, 2011). For psychological studies, however, it is often useful to provide a stricter operationalization of consonance, and so researchers commonly define consonance to their participants as the *pleasantness*, *beauty*, or *attractiveness* of a chord (e.g., Bowling & Purves, 2015; Bowling et al., 2018; Cousineau et al., 2012; McDermott et al., 2010, 2016).

In this paper we use the term “simultaneous” to restrict consideration to the notes within the chord, as opposed to sequential relationships between the chord and its musical context. Simultaneous and sequential consonance are sometimes termed *vertical* and *horizontal* consonance respectively, by analogy with the physical layout of the Western musical score (Parncutt & Hair, 2011). These kinds of chordal consonance may also be distinguished from “melodic” consonance, which refers to the intervals of a melody. For the remainder of this paper, the term “consonance” will be taken to imply “simultaneous consonance” unless specified otherwise.

Consonance and dissonance are often treated as two ends of a continuous scale, but some researchers treat the two as distinct phenomena (e.g., Parncutt & Hair, 2011). Under such formulations, consonance is typically treated as the perceptual correlate of harmonicity, and dissonance as the perceptual correlate of roughness (see *Consonance Theories*). Here we avoid this approach, and instead treat consonance and dissonance as antonyms.

## Consonance Theories

Here we review current theories of consonance perception. We pay particular attention to three classes of theories—periodicity/harmonicity, interference between partials, and culture—that we consider to be particularly well-supported by the empirical literature. We also discuss several related theories, including vocal similarity, fusion, and combination tones.

### Periodicity/Harmonicity

Human vocalizations are characterized by repetitive structure termed *periodicity*. This periodicity has several perceptual correlates, of which the most prominent is *pitch*. Broadly speaking, pitch corresponds to the waveform’s repetition rate, or *fundamental frequency*: Faster repetition corresponds to higher pitch.

Sound can be represented either in the time domain or in the frequency domain. In the time domain, periodicity manifests as repetitive waveform structure. In the frequency domain, periodicity manifests as *harmonicity*, a phenomenon where the sound’s frequency components are all integer multiples of the fundamental frequency.^3^ These integer-multiple frequencies are termed *harmonics*; a sound comprising a full set of integer multiples is termed a *harmonic series*. Each periodic sound constitutes a (possibly incomplete) harmonic series rooted on its fundamental frequency; conversely, every harmonic series (incomplete or complete) is periodic in its fundamental frequency. Harmonicity and periodicity are therefore essentially equivalent phenomena, and we will denote both by writing “periodicity/harmonicity.”

Humans rely on periodicity/harmonicity analysis to understand the natural environment and to communicate with others (e.g., Oxenham, 2018), but the precise mechanisms of this analysis remain unclear. The primary extant theories are time-domain *autocorrelation* theories and frequency-domain *pattern-matching* theories (de Cheveigné, 2005). Autocorrelation theories state that listeners detect periodicity by computing the signal’s correlation with a delayed version of itself as a function of delay time; peaks in the autocorrelation function correspond to potential fundamental frequencies (Balaguer-Ballester, Denham, & Meddis, 2008; Bernstein & Oxenham, 2005; Cariani, 1999; Cariani & Delgutte, 1996; de Cheveigné, 1998; Ebeling, 2008; Langner, 1997; Licklider, 1951; Meddis & Hewitt, 1991a, 1991b; Meddis & O’Mard, 1997; Slaney & Lyon, 1990; Wightman, 1973). Pattern-matching theories instead state that listeners infer fundamental frequencies by detecting harmonic patterns in the frequency domain (Bilsen, 1977; Cohen, Grossberg, & Wyse, 1995; Duifhuis, Willems, & Sluyter, 1982; Goldstein, 1973; Shamma & Klein, 2000; Terhardt, 1974; Terhardt, Stoll, & Seewann, 1982b). Both of these explanations have resisted definitive falsification, and it is possible that both mechanisms contribute to periodicity/harmonicity detection (de Cheveigné, 2005).

The prototypically consonant intervals of Western music tend to exhibit high periodicity/harmonicity. For example, octaves are typically performed as complex tones that approximate 2:1 frequency ratios, where every cycle of the lower-frequency waveform approximately coincides with a cycle of the higher-frequency waveform. The combined waveform therefore repeats approximately with a fundamental frequency equal to that of the lowest tone, which is as high a fundamental frequency as we could expect when combining two complex tones; we can therefore say that the octave has maximal periodicity. In contrast, the dissonant tritone cannot be easily approximated by a simple frequency ratio, and so its fundamental frequency (approximate or otherwise) must be much lower than that of the lowest tone. We therefore say that the tritone has relatively low periodicity.

It has correspondingly been proposed that periodicity/harmonicity determines consonance perception (Bidelman & Heinz, 2011; Boomsliter & Creel, 1961; Bowling & Purves, 2015; Bowling et al., 2018; Cousineau et al., 2012; Ebeling, 2008; Heffernan & Longtin, 2009; Lee, Skoe, Kraus, & Ashley, 2015; Lots & Stone, 2008; McDermott et al., 2010; Milne et al., 2016; Nordmark & Fahlén, 1988; Patterson, 1986; Spagnolo, Ushakov, & Dubkov, 2013; Stolzenburg, 2015; Terhardt, 1974; Ushakov, Dubkov, & Spagnolo, 2010).^4^ The nature of this potential relationship depends in large part on the unresolved issue of whether listeners detect periodicity/harmonicity using autocorrelation or pattern-matching (de Cheveigné, 2005), as well as other subtleties of auditory processing such as masking (Parncutt, 1989; Parncutt & Strasburger, 1994), octave invariance (Harrison & Pearce, 2018; Milne et al., 2016; Parncutt, 1988; Parncutt, Reisinger, Fuchs, & Kaiser, 2018), and nonlinear signal transformation (Lee et al., 2015; Stolzenburg, 2017). It is also unclear precisely how consonance develops from the results of periodicity/harmonicity detection; competing theories suggest that consonance is determined by the inferred fundamental frequency (Boomsliter & Creel, 1961; Stolzenburg, 2015), the absolute degree of harmonic template fit at the fundamental frequency (Bowling et al., 2018; Gill & Purves, 2009; Milne et al., 2016; Parncutt, 1989; Parncutt & Strasburger, 1994), the degree of template fit at the fundamental frequency relative to that at other candidate fundamental frequencies (Parncutt, 1988; Parncutt et al., 2018), or the degree of template fit as aggregated over all candidate fundamental frequencies (Harrison & Pearce, 2018). This variety of hypotheses is reflected in a diversity of computational models of musical periodicity/harmonicity perception (Ebeling, 2008; Gill & Purves, 2009; Harrison & Pearce, 2018; Lartillot, Toiviainen, & Eerola, 2008; Milne et al., 2016; Parncutt, 1988, 1989; Parncutt & Strasburger, 1994; Spagnolo et al., 2013; Stolzenburg, 2015). So far these models have only received limited empirical comparison (e.g., Stolzenburg, 2015).

It is clear why periodicity/harmonicity should be salient to human listeners: Periodicity/harmonicity detection is crucial for auditory scene analysis and for natural speech understanding (e.g., Oxenham, 2018). It is less clear why periodicity/harmonicity should be positively valenced, and hence associated with consonance. One possibility is that long-term exposure to vocal sounds (Schwartz et al., 2003) or Western music (McDermott et al., 2016) induces familiarity with periodicity/harmonicity, in turn engendering liking through the mere exposure effect (Zajonc, 2001). A second possibility is that the ecological importance of interpreting human vocalizations creates a selective pressure to perceive these vocalizations as attractive (Bowling et al., 2018).

### Interference Between Partials

Musical chords can typically be modeled as *complex tones*, superpositions of finite numbers of sinusoidal *pure tones* termed *partials*. Each partial is characterized by a frequency and an amplitude. It is argued that neighboring partials can interact to produce *interference* effects, with these interference effects subsequently being perceived as dissonance (Dillon, 2013; Helmholtz, 1863; Hutchinson & Knopoff, 1978; Kameoka & Kuriyagawa, 1969a, 1969b; Mashinter, 2006; Plomp & Levelt, 1965; Sethares, 1993; Vassilakis, 2001).

Pure-tone interference has two potential sources: *beating* and *masking*. Beating develops from the following mathematical identity for the addition of two equal-amplitude sinusoids:

$$\cos(2\pi f_{1}t)+\cos(2\pi f_{2}t)=2\cos\left(2\pi \bar{f}t\right)\cos\left(\pi \delta t\right)$$ 1

where *f*_1_, *f*_2_ are the frequencies of the original sinusoids (*f*_1_ > *f*_2_), $\bar{f}=(f_{1}+f_{2})/2$, δ = *f*_1_ − *f*_2_, and *t* denotes time. For sufficiently large frequency differences, listeners perceive the left hand side of Equation 1, corresponding to two separate pure tones at frequencies *f*_1_, *f*_2_. For sufficiently small frequency differences, listeners perceive the right hand side of Equation 1, corresponding to a tone of intermediate frequency $\bar{f}=(f_{1}+f_{2})/2$ modulated by a sinusoid of frequency $\delta /2=(f_{1}-f_{2})/2$. This modulation is perceived as amplitude fluctuation with frequency equal to the modulating sinusoid’s zero-crossing rate, *f*_1_ − *f*_2_. Slow amplitude fluctuation (c. 0.1–5 Hz) is perceived as a not unpleasant oscillation in loudness, but fast amplitude fluctuation (c. 20–30 Hz) takes on a harsh quality described as *roughness*. This roughness is thought to contribute to dissonance perception.

Masking describes situations where one sound obstructs the perception of another sound (e.g., Patterson & Green, 2012; Scharf, 1971). Masking in general is a complex phenomenon, but the mutual masking of pairs of pure tones can be approximated by straightforward mathematical models (Parncutt, 1989; Parncutt & Strasburger, 1994; Terhardt, Stoll, & Seewann, 1982a; Wang, Shen, Guo, Tang, & Hamade, 2013). These models embody long-established principles that masking increases with smaller frequency differences and with higher sound pressure level.

Beating and masking are both closely linked with the notion of *critical bands*. The notion of critical bands comes from modeling the cochlea as a series of overlapping *bandpass filters*, areas that are preferentially excited by spectral components within a certain frequency range (Zwicker, Flottorp, & Stevens, 1957). Beating typically only arises from spectral components localized to the same critical band (Daniel & Weber, 1997). The mutual masking of pure tones approximates a linear function of the number of critical bands separating them (termed *critical-band distance*), with additional masking occurring from pure tones within the same critical band that are unresolved by the auditory system (Terhardt et al., 1982a).

Beating and masking effects are both considerably stronger when two tones are presented diotically (to the same ear) rather than dichotically (to different ears; Buus, 1997; Grose, Buss, & Hall, 2012). This indicates that these phenomena depend, in large part, on physical interactions in the inner ear.

There is a long tradition of research relating beating to consonance, mostly founded on the work of Helmholtz (1863; Aures, 1985a, cited in Daniel & Weber, 1997; Hutchinson & Knopoff, 1978; Kameoka & Kuriyagawa, 1969a, 1969b; Mashinter, 2006; Parncutt et al., 2018; Plomp & Levelt, 1965; Sethares, 1993; Vassilakis, 2001).^5^ The general principle shared by this work is that consonance develops from the accumulation of roughness deriving from the beating of neighboring partials.

In contrast, the literature linking masking to consonance is relatively sparse. Huron (2001, 2002) suggests that masking induces dissonance because it reflects a compromised sensitivity to the auditory environment, with analogies in visual processing such as occlusion or glare. Aures (1984; cited in Parncutt, 1989) and Parncutt (1989; Parncutt & Strasburger, 1994) also state that consonance reduces as a function of masking. Unfortunately, these ideas have yet to receive much empirical validation; a difficulty is that beating and masking tend to happen in similar situations, making them difficult to disambiguate (Huron, 2001).

The kind of beating that elicits dissonance is achieved by small, but not too small, frequency differences between partials. With very small frequency differences, the beating becomes too slow to elicit dissonance (Hutchinson & Knopoff, 1978; Kameoka & Kuriyagawa, 1969a; Plomp & Levelt, 1965). The kind of masking that elicits dissonance is presumably also maximized by small, but not too small, frequency differences between partials. For moderately small frequency differences, the auditory system tries to resolve two partials, but finds it difficult on account of mutual masking, with this difficulty eliciting negative valence (Huron, 2001, 2002). For very small frequency differences, the auditory system only perceives one partial, which becomes purer as the two acoustic partials converge on the same frequency.

Musical sonorities can often be treated as combinations of *harmonic complex tones*, complex tones whose spectral frequencies follow a harmonic series. The interference experienced by a combination of harmonic complex tones depends on the fundamental frequencies of the complex tones. A particularly important factor is the ratio of these fundamental frequencies. Certain ratios, in particular the simple-integer ratios approximated by prototypically consonant musical chords, tend to produce partials that either completely coincide or are widely spaced, hence minimizing interference.

Interference between partials also depends on pitch height. A given frequency ratio occupies less critical-band distance as absolute frequency decreases, typically resulting in increased interference. This mechanism potentially explains why the same musical interval (e.g., the major third, 5:4) can sound consonant in high registers and dissonant in low registers.

It is currently unusual to distinguish beating and masking theories of consonance, as we have done above. Most previous work solely discusses beating and its psychological correlate, roughness (e.g., Cousineau et al., 2012; McDermott et al., 2010, 2016; Parncutt & Hair, 2011; Parncutt et al., 2018; Terhardt, 1984). However, we contend that the existing evidence does little to differentiate beating and masking theories, and that it would be premature to discard the latter in favor of the former. Moreover, we show later in this paper that computational models that address beating explicitly (e.g., Wang et al., 2013) seem to predict consonance worse than generic models of interference between partials (e.g., Hutchinson & Knopoff, 1978; Sethares, 1993; Vassilakis, 2001). For now, therefore, it seems wise to contemplate both beating and masking as potential contributors to consonance.

### Culture

Consonance may also be determined by a listener’s cultural background (Arthurs, Beeston, & Timmers, 2018; Guernsey, 1928; Johnson-Laird et al., 2012; Lundin, 1947; McDermott et al., 2016; McLachlan, Marco, Light, & Wilson, 2013; Omigie, Dellacherie, & Samson, 2017; Parncutt, 2006b; Parncutt & Hair, 2011). Several mechanisms for this effect are possible. Through the mere exposure effect (Zajonc, 2001), exposure to common chords in a musical style might induce familiarity and hence liking. Through classical conditioning, the co-occurrence of certain musical features (e.g., interference) with external features (e.g., the violent lyrics in death metal music, Olsen, Thompson, & Giblin, 2018) might also induce aesthetic responses to these musical features.

It remains unclear which musical features might become consonant through familiarity. One possibility is that listeners become familiar with acoustic phenomena such as periodicity/harmonicity (McDermott et al., 2016). A second possibility is that listeners internalize Western tonal structures such as diatonic scales (Johnson-Laird et al., 2012). Alternatively, listeners might develop a granular familiarity with specific musical chords (McLachlan et al., 2013).

### Other Theories

#### Vocal similarity

Vocal similarity theories hold that consonance derives from acoustic similarity to human vocalizations (e.g., Bowling & Purves, 2015; Bowling et al., 2018; Schwartz et al., 2003). A key feature of human vocalizations is periodicity/harmonicity, leading some researchers to operationalize vocal similarity as the latter (Gill & Purves, 2009). In such cases, vocal similarity theories may be considered a subset of periodicity/harmonicity theories. However, Bowling et al. (2018) additionally operationalize vocal similarity as the absence of frequency intervals smaller than 50 Hz, arguing that such intervals are rarely found in human vocalizations. Indeed, such intervals are negatively associated with consonance; however, this phenomenon can also be explained by interference minimization. To our knowledge, no studies have shown that vocal similarity contributes to consonance through paths other than periodicity/harmonicity and interference. We therefore do not evaluate vocal similarity separately from interference and periodicity/harmonicity.

#### Fusion

Stumpf (1890, 1898) proposed that consonance derives from *fusion*, the perceptual merging of multiple harmonic complex tones. The substance of this hypothesis depends on the precise definition of fusion. Some researchers have operationalized fusion as *perceptual indiscriminability*, that is, an inability to identify the constituent tones of a sonority (DeWitt & Crowder, 1987; McLachlan et al., 2013). This was encouraged by Stumpf’s early experiments investigating how often listeners erroneously judged tone pairs as single tones (DeWitt & Crowder, 1987; Schneider, 1997). Subsequently, however, Stumpf wrote that fusion should not be interpreted as indiscriminability but rather as the formation of a coherent whole, with the sophisticated listener being able to attend to individual chord components at will (Schneider, 1997). Stumpf later wrote that he was unsure whether fusion truly caused consonance; instead, he suggested that fusion and consonance might both stem from harmonicity recognition (Plomp & Levelt, 1965; Schneider, 1997).

Following Stumpf, several subsequent studies have investigated the relationship between fusion and consonance, but with mixed findings. Guernsey (1928) and DeWitt and Crowder (1987) tested fusion by playing participants different dyads and asking how many tones these chords contained. In both studies, prototypically consonant musical intervals (octaves, perfect fifths) were most likely to be confused for single tones, supporting a link between consonance and fusion. McLachlan et al. (2013) instead tested fusion with a pitch-matching task, where each trial cycled between a target chord and a probe tone, and participants were instructed to manipulate the probe tone until it matched a specified chord tone (lowest, middle, or highest). Pitch-matching accuracy increased for prototypically consonant chords, suggesting (contrary to Stumpf’s claims) that consonance was *inversely* related to fusion. It is difficult to conclude much about Stumpf’s claims from these studies, partly because different studies have yielded contradictory results, and partly because none of these studies tested for *causal* effects of fusion on consonance, as opposed to consonance and fusion both being driven by a common factor of periodicity/harmonicity.

#### Combination tones

*Combination tones* are additional spectral components introduced by nonlinear sound transmission in the ear’s physical apparatus (e.g., Parncutt, 1989; Smoorenburg, 1972; Wever, Bray, & Lawrence, 1940). For example, two pure tones of frequencies *f*_1_, *f*_2_ : *f*_1_ < *f*_2_ can elicit combination tones including the *simple difference tone* (*f* = *f*_2_ − *f*_1_) and the *cubic difference tone* (*f* = 2*f*_1_ − *f*_2_; Parncutt, 1989; Smoorenburg, 1972).

Combination tones were once argued to be an important mechanism for pitch perception, reinforcing a complex tone’s fundamental frequency and causing it to be perceived even when not acoustically present (e.g., Fletcher, 1924; see Parncutt, 1989). Combination tones were also argued to have important implications for music perception, explaining phenomena such as chord roots and perceptual consonance (Hindemith, 1945; Krueger, 1910; Tartini, 1754, cited in Parncutt, 1989). However, subsequent research showed that the missing fundamental persisted even when the difference tone was removed by acoustic cancellation (Schouten, 1938, described in Plomp, 1965), and that, in any case, difference tones are usually too quiet to be audible for typical speech and music listening (Plomp, 1965). We therefore do not consider combination tones further.

#### Loudness and sharpness

Aures (1985a, 1985b) describes four aspects of sensory consonance: *tonalness*, *roughness*, *loudness*, and *sharpness*. Tonalness is a synonym for periodicity/harmonicity, already discussed as an important potential contributor to consonance. Roughness is an aspect of interference, also an important potential contributor to consonance. Loudness is the perceptual correlate of a sound’s energy content; sharpness describes the energy content of high spectral frequencies. Historically, loudness and sharpness have received little attention in the study of musical consonance, perhaps because music theorists and psychologists have primarily been interested in the consonance of transposition-invariant and loudness-invariant structures such as pitch-class sets, for which loudness and sharpness are undefined. We do not consider these phenomena further.

#### Evenness

The constituent notes of a musical chord can be represented as points on a *pitch line* or a *pitch-class circle* (e.g., Tymoczko, 2016). The *evenness* of the resulting distribution can be characterized in various ways, including the difference in successive interval sizes (Cook, 2009, 2017; Cook & Fujisawa, 2006), the difference between the largest and smallest interval sizes (Parncutt et al., 2018), and the standard deviation of interval sizes (Parncutt et al., 2018). In the case of Cook’s (2009, 2017, Cook & Fujisawa, 2006) models, each chord note is expanded into a harmonic complex tone, and pitch distances are computed between the resulting partials; in the other cases, pitch distances are computed between fundamental frequencies, presumably as inferred through periodicity/harmonicity detection.

Evenness may contribute negatively to consonance. When a chord contains multiple intervals of the same size, these intervals may become confusable and impede perceptual organization, hence decreasing consonance (Cook, 2009, 2017; Cook & Fujisawa, 2006; Meyer, 1956). For example, a major triad in pitch-class space contains the intervals of a major third, a minor third, and a perfect fourth, and each note of the triad participates in a unique pair of these intervals, one connecting it to the note above, and one connecting it to the note below. In contrast, an augmented triad contains only intervals of a major third, and so each note participates in an identical pair of intervals. Correspondingly, the individual notes of the augmented triad may be considered less distinctive than those of the major triad.

Evenness may also contribute positively, but indirectly, to consonance. Spacing harmonics evenly on a critical-band scale typically reduces interference, thereby increasing consonance (see, e.g., Huron & Sellmer, 1992; Plomp & Levelt, 1965). Evenness also facilitates efficient voice leading, and therefore may contribute positively to sequential consonance (Parncutt et al., 2018; Tymoczko, 2011).

Evenness is an interesting potential contributor to consonance, but so far it has received little empirical testing. We do not consider it to be sufficiently well-supported to include in this paper’s analyses, but we encourage future empirical research on the topic.

## Current Evidence

Evidence for disambiguating different theories of consonance perception can be organized into three broad categories: *stimulus effects*, *listener effects*, and *composition effects*. We review each of these categories in turn, and summarize our conclusions in Table 1.[Table-anchor tbl1]

### Stimulus Effects

We begin by discussing *stimulus effects*, ways in which consonance perception varies as a function of the stimulus.

#### Tone spectra

A chord’s consonance depends on the spectral content of its tones. With harmonic tone spectra, peak consonance is observed when the fundamental frequencies are related by simple frequency ratios (e.g., Stolzenburg, 2015). With pure tone spectra, these peaks at integer ratios disappear, at least for musically untrained listeners (Kaestner, 1909; Plomp & Levelt, 1965). With inharmonic tone spectra, the peaks at integer ratios are replaced by peaks at ratios determined by the inharmonic spectra (Geary, 1980; Pierce, 1966; Sethares, 2005).^6^ The consonance of harmonic tone combinations can also be increased by selectively deleting harmonics responsible for interference (Vos, 1986), though Nordmark and Fahlén (1988) report limited success with this technique.

Interference theories clearly predict these effects of tone spectra on consonance (for harmonic and pure tones, see Plomp & Levelt, 1965; for inharmonic tones, see Sethares, 1993, 2005). In contrast, neither periodicity/harmonicity nor cultural theories clearly predict these phenomena. This suggests that interference does indeed contribute toward consonance perception.

#### Pitch height

A given interval ratio typically appears less consonant if it appears at low frequencies (Plomp & Levelt, 1965). Interference theories predict this phenomenon by relating consonance to pitch distance on a critical-bandwidth scale; a given ratio corresponds to a smaller critical-bandwidth distance if it appears at lower frequencies (Plomp & Levelt, 1965). In contrast, neither periodicity/harmonicity nor cultural theories predict this sensitivity to pitch height.

#### Dichotic presentation

Interference between partials is thought to take place primarily within the inner ear. Correspondingly, the interference of a given pair of pure tones can be essentially eliminated by dichotic presentation, where each tone is presented to a separate ear. Periodicity/harmonicity detection, meanwhile, is thought to be a central process that combines information from both ears (Cramer & Huggins, 1958; Houtsma & Goldstein, 1972). Correspondingly, the contribution of periodicity/harmonicity detection to consonance perception should be unaffected by dichotic presentation.

Bidelman and Krishnan (2009) report consonance judgments for dichotically presented pairs of complex tones. Broadly speaking, participants continued to differentiate prototypically consonant and dissonant intervals, suggesting that interference is insufficient to explain consonance. Unexpectedly, however, the tritone and perfect fourth received fairly similar consonance ratings. This finding needs to be explored further.

Subsequent studies have investigated the effect of dichotic presentation on consonance judgments for pairs of pure tones (Cousineau et al., 2012; McDermott et al., 2010, 2016). These studies show that dichotic presentation reliably increases the consonance of small pitch intervals, in particular major and minor seconds, as predicted by interference theories. This would appear to support interference theories of consonance, though it is unclear whether these effects generalize to the complex tone spectra of real musical instruments.

#### Familiarity

McLachlan et al. (2013, Experiment 2) trained nonmusicians to perform a pitch-matching task on two-note chords. After training, participants judged chords from the training set as more consonant than novel chords. These results could be interpreted as evidence that consonance is positively influenced by exposure, consistent with the mere exposure effect, and supporting a cultural theory of consonance. However, the generalizability of this effect has yet to be confirmed.

#### Chord structure

Western listeners consider certain chords (e.g., the major triad) to be more consonant than others (e.g., the augmented triad). It is possible to test competing theories of consonance by operationalizing the theories as computational models and testing their ability to predict consonance judgments.

Unfortunately, studies using this approach have identified conflicting explanations for consonance:

1 Interference (Hutchinson & Knopoff, 1978);
2 Interference and additional unknown factors (Vassilakis, 2001);
3 Interference and cultural knowledge (Johnson-Laird et al., 2012);
4 Periodicity/harmonicity (Stolzenburg, 2015);
5 Periodicity/harmonicity and interference (Marin, Forde, Gingras, & Stewart, 2015);
6 Interference and sharpness (Lahdelma & Eerola, 2016);
7 Vocal similarity (Bowling et al., 2018).

These contradictions may often be attributed to methodological problems:

1 Different studies test different theories, and rarely test more than two theories simultaneously.
2 Stimulus sets are often too small to support reliable inferences.^7^
3 Stolzenburg (2015) evaluates models using pairwise correlations, implicitly assuming that only one mechanism (e.g., periodicity/harmonicity, interference) determines consonance. Multiple regression would be necessary to capture multiple simultaneous mechanisms.
4 The stimulus set of Marin et al. (2015) constitutes 12 dyads each transposed four times; the conditional dependencies between transpositions are not accounted for in the linear regressions, inflating Type I error.
5 Johnson-Laird et al. (2012) do not report coefficients or *p* values for their fitted regression models; they do report hierarchical regression statistics, but these statistics do not test their primary research question, namely whether interference and cultural knowledge *simultaneously* contribute to consonance.
6 The audio-based periodicity/harmonicity model used by Lahdelma and Eerola (2016) fails when applied to complex stimuli such as chords (see the Perceptual Analyses section).

These methodological problems and contradictory findings make it difficult to generalize from this literature.

### Listener Effects

We now discuss *listener effects*, ways in which consonance perception varies as a function of the listener.

#### Western listeners

McDermott et al. (2010) tested competing theories of consonance perception using an individual-differences approach. They constructed three psychometric measures, testing: (a) *Interference preferences*, operationalized by playing listeners pure-tone dyads and subtracting preference ratings for dichotic presentation (one tone in each ear) from ratings for diotic presentation (both tones in both ears); (b) *Periodicity/harmonicity preferences*, operationalized by playing listeners subsets of a harmonic complex tone and subtracting preference ratings for the original version from ratings for a version with perturbed harmonics; (c) *Consonance preferences*, operationalized by playing listeners 14 musical chords, and subtracting preference ratings for the globally least-preferred chords from the globally most-preferred chords.

Consonance preferences correlated with periodicity/harmonicity preferences but not with interference preferences. This suggests that consonance may be driven by periodicity/harmonicity, not interference. However, these findings must be considered preliminary given the limited construct validation of the three psychometric measures. Future work must examine whether these measures generalize to a wider range of stimulus manipulations and response paradigms.

#### Congenital amusia

Congenital amusia is a lifelong cognitive disorder characterized by difficulties in performing simple musical tasks (Ayotte, Peretz, & Hyde, 2002; Stewart, 2011). Using the individual-differences tests of McDermott et al. (2010) (see the Western listeners section), Cousineau et al. (2012) found that amusics exhibited no aversion to traditionally dissonant chords, normal aversion to interference, and an inability to detect periodicity/harmonicity. Because the aversion to interference did not transfer to dissonant chords, Cousineau et al. (2012) concluded that interference is irrelevant to consonance perception. However, Marin et al. (2015) subsequently identified small but reliable preferences for consonance in amusics, and showed with regression analyses that these preferences were driven by interference, whereas nonamusic preferences were driven by both interference and periodicity/harmonicity. This discrepancy between Cousineau et al. (2012) and Marin et al. (2015) needs further investigation.

#### Non-Western listeners

Cross-cultural research into consonance perception has identified high similarity between the consonance judgments of Western and Japanese listeners (Butler & Daston, 1968), but low similarity between Western and Indian listeners (Maher, 1976), and between Westerners and native Amazonians from the Tsimane’ society (McDermott et al., 2016). Exploring these differences further, McDermott et al. (2016) found that Tsimane’ and Western listeners shared an aversion to interference and an ability to perceive periodicity/harmonicity, but, unlike Western listeners, the Tsimane’ had no *preference* for periodicity/harmonicity.

These results suggest that cultural exposure significantly affects consonance perception. The results of McDermott et al. (2016) additionally suggest that this effect of cultural exposure may be mediated by changes in preference for periodicity/harmonicity.

#### Infants

Consonance perception has been demonstrated in toddlers (Di Stefano et al., 2017), 6-month-old infants (Crowder, Reznick, & Rosenkrantz, 1991; Trainor & Heinmiller, 1998), 4-month-old infants (Trainor, Tsang, & Cheung, 2002; Zentner & Kagan, 1998), 2-month-old infants (Trainor et al., 2002), and newborn infants (Masataka, 2006; Perani et al., 2010; Virtala, Huotilainen, Partanen, Fellman, & Tervaniemi, 2013). Masataka (2006) additionally found preserved consonance perception in newborn infants with deaf parents. These results suggest that consonance perception does not solely depend on cultural exposure.

A related question is whether infants *prefer* consonance to dissonance. Looking-time paradigms address this question, testing whether infants preferentially look at consonant or dissonant sound sources (Crowder et al., 1991; Masataka, 2006; Plantinga & Trehub, 2014; Trainor & Heinmiller, 1998; Trainor et al., 2002; Zentner & Kagan, 1998). With the exception of Plantinga and Trehub (2014), these studies each report detecting consonance preferences in infants. However, Plantinga and Trehub (2014) failed to replicate several of these results, and additionally question the validity of looking-time paradigms, noting that looking times may be confounded by features such as familiarity and comprehensibility. These problems may partly be overcome by physical play-based paradigms (e.g., Di Stefano et al., 2017), but such paradigms are unfortunately only applicable to older infants.

In conclusion, therefore, it seems that young infants perceive some aspects of consonance, but it is unclear whether they prefer consonance to dissonance. These conclusions provide tentative evidence that consonance perception is not solely cultural.

#### Animals

Animal studies could theoretically provide compelling evidence for noncultural theories of consonance. If animals were to display sensitivity or preference for consonance despite zero prior musical exposure, this would indicate that consonance could not be fully explained by cultural learning.

Most studies of consonance perception in animals fall into two categories: *discrimination* studies and *preference* studies (see Toro & Crespo-Bojorque, 2017 for a review). Discrimination studies investigate whether animals can be taught to discriminate consonance from dissonance in unfamiliar sounds. Preference studies investigate whether animals prefer consonance to dissonance.

Discrimination studies have identified consonance discrimination in several nonhuman species, but methodological issues limit interpretation of their findings. Experiment 5 of Hulse, Bernard, and Braaten (1995) suggests that starlings may be able to discriminate consonance from dissonance, but their stimulus set contains just four chords. Experiment 2 of Izumi (2000) suggests that Japanese monkeys may be able to discriminate consonance from dissonance, but this study likewise relies on just four chords at different transpositions. Watanabe, Uozumi, and Tanaka (2005) claim to show consonance discrimination in Java sparrows, but the sparrows’ discriminations can also be explained by interval-size judgments.^8^ Conversely, studies of pigeons (Brooks & Cook, 2010) and rats (Crespo-Bojorque & Toro, 2015) have failed to show evidence of consonance discrimination (but see also Borchgrevink, 1975).^9^

Preference studies have identified consonance preferences in several nonhuman animals. Using stimuli from a previous infant consonance study (Zentner & Kagan, 1998), Chiandetti and Vallortigara (2011) found that newly hatched domestic chicks spent more time near consonant sound sources than dissonant sound sources. Sugimoto et al. (2010) gave an infant chimpanzee the ability to select between consonant and dissonant two-part melodies, and found that the chimpanzee preferentially selected consonant melodies. However, these studies have yet to be replicated, and both rely on borderline *p* values (*p* = .03). Other studies have failed to demonstrate consonance preferences in Campbell’s monkeys (Koda et al., 2013) or cotton-top tamarins (McDermott & Hauser, 2004).

These animal studies provide an important alternative perspective on consonance perception. However, recurring problems with these studies include small stimulus sets, small sample sizes, and a lack of replication studies. Future work should address these problems.

### Composition Effects

Here we consider how compositional practice may provide evidence for the psychological mechanisms underlying consonance perception.

#### Musical scales

A *scale* divides an octave into a set of pitch classes that can subsequently be used to generate musical material. Scales vary cross-culturally, but certain cross-cultural similarities between scales suggest common perceptual biases.

Gill and Purves (2009) argue that scale construction is biased toward harmonicity maximization, and explain harmonicity maximization as a preference for vocal-like sounds. They introduce a computational model of harmonicity, which successfully recovers several important scales in Arabic, Chinese, Indian, and Western music. However, they do not test competing consonance models, and admit that their results may also be explained by interference minimization.

Gamelan music and Thai classical music may help distinguish periodicity/harmonicity from interference. Both traditions use inharmonic scales whose structures seemingly reflect the inharmonic spectra of their percussion instruments (Sethares, 2005). Sethares provides computational analyses relating these scales to interference minimization; periodicity/harmonicity, meanwhile, offers no obvious explanation for these scales.^10^ These findings suggest that interference contributes cross-culturally to consonance perception.

#### Manipulation of interference

Western listeners typically perceive interference as unpleasant, but various other musical cultures actively promote it. Interference is a key feature of the Middle Eastern *mijwiz*, an instrument comprising two blown pipes whose relative tunings are manipulated to induce varying levels of interference (Vassilakis, 2005). Interference is also promoted in the vocal practice of *beat diaphony*, or *Schwebungsdiaphonie*, where two simultaneous voice parts sing in close intervals such as seconds. Beat diaphony can be found in various musical traditions, including music from Lithuania (Ambrazevičius, 2017; Vyčinienė, 2002), Papua New Guinea (Florian, 1981), and Bosnia (Vassilakis, 2005). In contrast to Western listeners, individuals from these traditions seem to perceive the resulting sonorities as consonant (Florian, 1981). These cross-cultural differences indicate that the aesthetic valence of interference is, at least in part, culturally determined.

#### Chord spacing (Western music)

In Western music, chords seem to be spaced to minimize interference, most noticeably by avoiding small intervals in lower registers but permitting them in higher registers (Huron & Sellmer, 1992; McGowan, 2011; Plomp & Levelt, 1965). Periodicity theories of consonance provide no clear explanation for this phenomenon.

#### Chord prevalences (Western music)

Many theorists have argued that consonance played an integral role in determining Western compositional practice (e.g., Dahlhaus, 1990; Hindemith, 1945; Rameau, 1722). If so, it should be possible to test competing theories of consonance by examining their ability to predict compositional practice.

Huron (1991) analyzed prevalences of different intervals within 30 polyphonic keyboard works by J. S. Bach, and concluded that they reflected dual concerns of minimizing interference and minimizing tonal fusion. Huron argued that interference was minimized on account of its negative aesthetic valence, whereas tonal fusion was minimized to maintain perceptual independence of the different voices.

Parncutt et al. (2018) tabulated chord types in seven centuries of vocal polyphony, and related their occurrence rates to several formal models of diatonicity, interference, periodicity/harmonicity, and evenness. Most models correlated significantly with chord occurrence rates, with fairly stable coefficient estimates across centuries. These results suggest that multiple psychological mechanisms contribute to consonance.

However, these findings must be treated as tentative, for the following reasons: (a) The parameter estimates have low precision due to the small sample sizes (12 dyads in Huron, 1991; 19 triads in Parncutt et al., 2018)^11^; (b) The pairwise correlations reported in Parncutt et al. (2018) cannot capture effects of multiple concurrent mechanisms (e.g., periodicity/harmonicity and interference).

### Discussion

Table 1 summarizes the evidence contributed by these diverse studies. We now use this evidence to reevaluate some claims in the recent literature.

#### Role of periodicity/harmonicity

Recent work has claimed that consonance is primarily determined by periodicity/harmonicity, with the role of periodicity/harmonicity potentially moderated by musical background (Cousineau et al., 2012; McDermott et al., 2010, 2016). In our view, a significant contribution of periodicity/harmonicity to consonance is indeed supported by the present literature, in particular by individual-differences research and congenital amusia research (see Table 1). A moderating effect of musical background also seems likely, on the basis of cross-cultural variation in music perception and composition. However, quantitative descriptions of these effects are missing: It is unclear what proportion of consonance may be explained by periodicity/harmonicity, and it is unclear how sensitive consonance is to cultural exposure.

#### Role of interference

Recent work has also claimed that consonance is independent of interference (Bowling & Purves, 2015; Bowling et al., 2018; Cousineau et al., 2012; McDermott et al., 2010, 2016). In our view, the wider literature is inconsistent with this claim (see Table 1). The main evidence against interference comes from the individual-differences study of McDermott et al. (2010), but this evidence is counterbalanced by several positive arguments for interference, including studies of tone spectra, pitch height, chord voicing in Western music, scale tunings in Gamelan music and Thai classical music, and cross-cultural manipulation of interference for expressive effect.

#### Role of culture

Cross-cultural studies of music perception and composition make it clear that culture contributes to consonance perception (see Table 1). The mechanisms of this effect remain unclear, however: Some argue that Western listeners internalize codified conventions of Western harmony (Johnson-Laird et al., 2012), whereas others argue that Westerners simply learn aesthetic preferences for periodicity/harmonicity (McDermott et al., 2016). These competing explanations have yet to be tested.

#### Conclusions

We conclude that consonance perception in Western listeners is likely to be driven by multiple psychological mechanisms, including interference, periodicity/harmonicity, and cultural background (see Table 1). This conclusion is at odds with recent claims that interference does not contribute to consonance perception (Cousineau et al., 2012; McDermott et al., 2010, 2016). In the rest of this paper, we therefore examine our proposition empirically, computationally modeling large datasets of consonance judgments and music compositions.

## Computational Models

**Figure 1 fig1:**
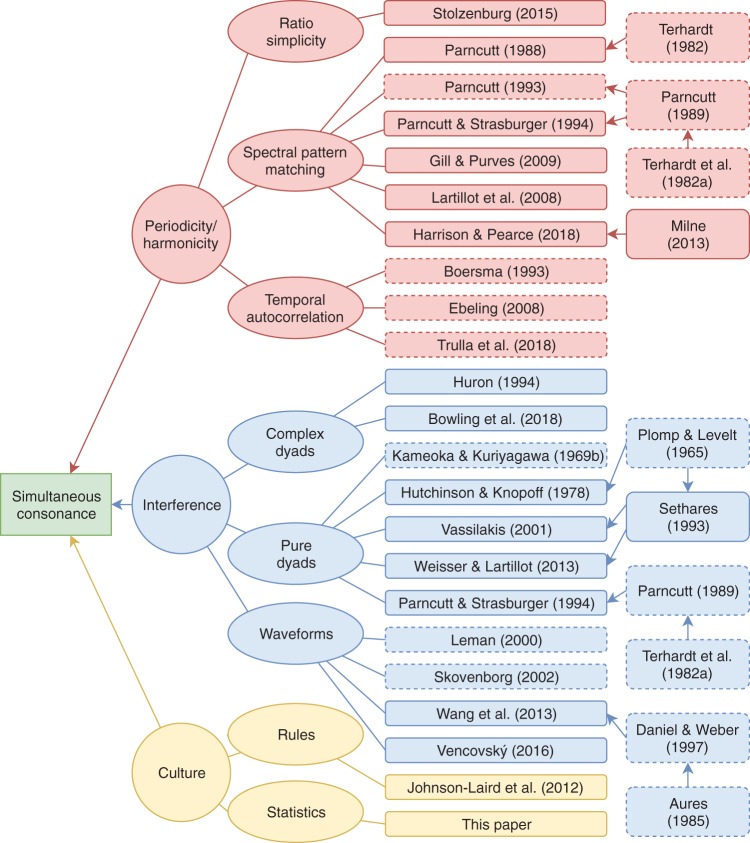
Consonance models organized by psychological theory and modeling approach. Dashed borders indicate models not evaluated in our empirical analyses. Arrows denote model revisions.

We begin by reviewing prominent computational models of consonance from the literature, organizing them by psychological theory and by modeling approach (see Figure 1).[Fig-anchor fig1]

### Periodicity/Harmonicity: Ratio Simplicity

Chords tend to be more periodic when their constituent tones are related by simple frequency ratios. Ratio simplicity can therefore provide a proxy for periodicity/harmonicity. Previous research has formalized ratio simplicity in various ways, with the resulting measures predicting the consonance of just-tuned chords fairly well (e.g., Euler, 1739; Geer, Levelt, & Plomp, 1962; Levelt, van de Geer, & Plomp, 1966; Schellenberg & Trehub, 1994).^12^ Unfortunately, these measures generally fail to predict consonance for chords that are not just-tuned. A particular problem is disproportionate sensitivity to small tuning deviations: For example, an octave stretched by 0.001% still sounds consonant, despite corresponding to a very complex frequency ratio (200,002:100,000). However, Stolzenburg (2015) provides an effective solution to this by introducing a preprocessing step where each note is adjusted to maximize ratio simplicity with respect to the bass note. These adjustments are not permitted to change the interval size by more than 1.1%. Stolzenburg argues that such adjustments are reasonable given human perceptual inaccuracies in pitch discrimination. Having expressed each chord frequency as a fractional multiple of the bass frequency, ratio simplicity is then computed as the lowest common multiple of the fractions’ denominators. Stolzenburg terms this expression *relative periodicity*, and notes that, assuming harmonic tones, relative periodicity corresponds to the chord’s overall period length divided by the bass tone’s period length. Relative periodicity values are then postprocessed with logarithmic transformation and smoothing to produce the final model output (see Stolzenburg, 2015 for details).

### Periodicity/Harmonicity: Spectral Pattern Matching

Spectral pattern-matching models of consonance follow directly from spectral pattern-matching theories of pitch perception (see the Consonance Theories section). These models operate in the frequency domain, searching for spectral patterns characteristic of periodic sounds.

#### Terhardt (1982); Parncutt (1988)

Terhardt (1982) and Parncutt (1988) both frame consonance in terms of chord-root perception. In Western music theory, the chord root is a pitch class summarizing a chord’s tonal content, which (according to Terhardt and Parncutt) arises through pattern-matching processes of pitch perception. Consonance arises when a chord has a clear root; dissonance arises from root ambiguity.

Both Terhardt’s (1982) and Parncutt’s (1988) models use harmonic templates quantized to the Western 12-tone scale, with the templates represented as octave-invariant pitch class sets. Each pitch class receives a numeric weight, quantifying how well the chord’s pitch classes align with a harmonic template rooted on that pitch class. These weights preferentially reward coincidence with primary harmonics such as the octave, perfect fifth, and major third.^13^ The chord root is estimated as the pitch class with the greatest weight; root ambiguity is then operationalized by dividing the total weight by the maximum weight. According to Terhardt and Parncutt, root ambiguity should then negatively predict consonance.

#### Parncutt (1989); Parncutt and Strasburger (1994)

Parncutt’s (1989) model constitutes a musical revision of Terhardt et al.’s (1982a) pitch perception algorithm. Parncutt and Strasburger’s (1994) model, in turn, represents a slightly updated version of Parncutt’s (1989) model.

Like Parncutt’s (1988) model, Parncutt’s (1989) model formulates consonance in terms of pattern-matching pitch perception. As in Parncutt (1988), the algorithm works by sweeping a harmonic template across an acoustic spectrum, seeking locations where the template coincides well with the acoustic input; consonance is elicited when the location of best fit is unambiguous. However, Parncutt’s (1989) algorithm differs from Parncutt (1988) in several important ways: (a) Chord notes are expanded into their implied harmonics; (b) Psychoacoustic phenomena such as hearing thresholds, masking, and audibility saturation are explicitly modeled; (c) The pattern-matching process is no longer octave-invariant.

Parncutt (1989) proposes two derived measures for predicting consonance: *pure tonalness* and *complex tonalness*.^14^ Pure tonalness describes the extent to which the input spectral components are audible, after accounting for hearing thresholds and masking. Complex tonalness describes the audibility of the strongest virtual pitch percept. The former may be considered a interference model, the latter a periodicity/harmonicity model.

Parncutt and Strasburger (1994) describe an updated version of Parncutt’s (1989) algorithm. The underlying principles are the same, but certain psychoacoustic details differ, such as the calculation of pure-tone audibility thresholds and the calculation of pure-tone height. We evaluate this updated version here.

Parncutt (1993) presents a related algorithm for modeling the perception of octave-spaced tones (also known as Shepard tones). Because octave-spaced tones are uncommon in Western music, we do not evaluate the model here.

#### Gill and Purves (2009)

Gill and Purves (2009) present a pattern-matching periodicity/harmonicity model which they apply to various two-note chords. They assume just tuning, which allows them to compute each chord’s fundamental frequency as the greatest common divisor of the two tones’ frequencies. They then construct a hypothetical harmonic complex tone rooted on this fundamental frequency, and calculate what proportion of this tone’s harmonics are contained within the spectrum of the original chord. This proportion forms their periodicity/harmonicity measure. This approach has been shown to generalize well to three- and four-note chords (Bowling et al., 2018). However, the model’s cognitive validity is limited by the fact that, unlike human listeners, it is very sensitive to small deviations from just tuning or harmonic tone spectra.

#### Peeters et al. (2011); Bogdanov et al. (2013); Lartillot et al. (2008)

Several prominent audio analysis toolboxes—the Timbre Toolbox (Peeters et al., 2011), Essentia (Bogdanov et al., 2013), and MIRtoolbox (Lartillot et al., 2008)—contain inharmonicity measures. Here we examine their relevance for consonance modeling.

The inharmonicity measure in the Timbre Toolbox (Peeters et al., 2011) initially seems relevant for consonance modeling, being calculated by summing each partial’s deviation from harmonicity. However, the algorithm’s preprocessing stages are clearly designed for single tones rather than tone combinations. Each input spectrum is preprocessed to a harmonic spectrum, slightly deformed by optional stretching; this may be a reasonable approximation for single tones, but it is inappropriate for tone combinations. We therefore do not consider this model further.

Essentia (Bogdanov et al., 2013) contains an inharmonicity measure defined similarly to the Timbre Toolbox (Peeters et al., 2011). As with the Timbre Toolbox, this feature is clearly intended for single tones rather than tone combinations, and so we do not consider it further.

MIRtoolbox (Lartillot et al., 2008) contains a more flexible inharmonicity measure. First, the fundamental frequency is estimated using autocorrelation and peak-picking; inharmonicity is then estimated by applying a sawtooth filter to the spectrum, with troughs corresponding to integer multiples of the fundamental frequency, and then integrating the result. This measure seems more likely to capture inharmonicity in musical chords, and indeed it has been recently used in consonance perception research (Lahdelma & Eerola, 2016). However, systematic validations of this measure are lacking.

#### Milne (2013); Harrison and Pearce (2018)

Milne (2013) presents a periodicity/harmonicity model that operates on pitch-class spectra (see also Milne et al., 2016). The model takes a pitch-class set as input, and expands all tones to idealized harmonic spectra. These spectra are superposed additively, and then blurred by convolution with a Gaussian distribution, mimicking perceptual uncertainty in pitch processing. The algorithm then sweeps a harmonic template over the combined spectrum, calculating the cosine similarity between the template and the combined spectrum as a function of the template’s fundamental frequency. The frequency eliciting the maximal cosine similarity is identified as the fundamental frequency, and the resulting cosine similarity is taken as the periodicity/harmonicity estimate.

Harrison and Pearce (2018) suggest that picking just one fundamental frequency may be inappropriate for larger chords, where listeners may instead infer several candidate fundamental frequencies. They therefore treat the cosine-similarity profile as a probability distribution, and define periodicity/harmonicity as the Kullback-Leibler divergence to this distribution from a uniform distribution. The resulting measure can be interpreted as the information-theoretic uncertainty of the pitch-estimation process.

### Periodicity/Harmonicity: Temporal Autocorrelation

Temporal autocorrelation models of consonance follow directly from autocorrelation theories of pitch perception (see *Consonance Theories*). These models operate in the time domain, looking for time lags at which the signal correlates with itself: High autocorrelation implies periodicity and hence consonance.

#### Boersma (1993)

Boersma’s (1993) autocorrelation algorithm can be found in the popular phonetics software Praat. The algorithm tracks the fundamental frequency of an acoustic input over time, and operationalizes periodicity as the *harmonics-to-noise ratio*, the proportion of power contained within the signal’s periodic component. Marin et al. (2015) found that this algorithm had some power to predict the relative consonance of different dyads. However, the details of the algorithm lack psychological realism, having been designed to solve an engineering problem rather than to simulate human perception. This limits the algorithm’s appeal as a consonance model.

#### Ebeling (2008)

Ebeling’s (2008) autocorrelation model estimates the consonance of pure-tone intervals. Incoming pure tones are represented as sequences of discrete pulses, reflecting the neuronal rate coding of the peripheral auditory system. These pulse sequences are additively superposed to form a composite pulse sequence, for which the autocorrelation function is computed. The *generalized coincidence function* is then computed by integrating the squared autocorrelation function over a finite positive range of time lags. Applied to pure tones, the generalized coincidence function recovers the traditional hierarchy of intervallic consonance, and mimics listeners in being tolerant to slight mistunings. Ebeling presents this as a positive result, but it is inconsistent with Plomp and Levelt’s (1965) observation that, after accounting for musical training, pure tones do not exhibit the traditional hierarchy of intervallic consonance. It remains unclear whether the model would successfully generalize to larger chords or to complex tones.

#### Trulla, Stefano, and Giuliani (2018)

Trulla et al.’s (2018) model uses *recurrence quantification analysis* to model the consonance of pure-tone intervals. Recurrence quantification analysis performs a similar function to autocorrelation analysis, identifying time lags at which waveform segments repeat themselves. Trulla et al. (2018) use this technique to quantify the amount of repetition within a waveform, and show that repetition is maximized by traditionally consonant frequency ratios, such as the just-tuned perfect fifth (3:2). The algorithm constitutes an interesting new approach to periodicity/harmonicity detection, but one that lacks much cognitive or neuroscientific backing. As with Ebeling (2008), it is also unclear how well the algorithm generalizes to larger chords or to different tone spectra, and the validation suffers from the same problems described above for Ebeling’s model.

#### Summary

Autocorrelation is an important candidate mechanism for consonance perception. However, autocorrelation consonance models have yet to be successfully generalized outside simple tone spectra and two-note intervals. We therefore do not evaluate these models in the present work, but we look forward to future research in this area (see, e.g., Tabas et al., 2017).

### Interference: Complex Dyads

Complex-dyad models of interference search chords for complex dyads known to elicit interference. These models are typically hand-computable, making them well-suited to quick consonance estimation.

#### Huron (1994)

Huron (1994) presents a measure termed *aggregate dyadic consonance*, which characterizes the consonance of a pitch-class set by summing consonance ratings for each pitch-class interval present in the set. These consonance ratings are derived by aggregating perceptual data from previous literature.

Huron (1994) originally used aggregate dyadic consonance to quantify a scale’s ability to generate consonant intervals. Parncutt et al. (2018) subsequently applied the model to musical chords, and interpreted the output as an interference measure. The validity of this approach rests on the assumption that interference is additively generated by pairwise interactions between spectral components; a similar assumption is made by pure-dyad interference models (see the Interference: Pure Dyads section). A further assumption is that Huron’s dyadic consonance ratings solely reflect interference, not (e.g.) periodicity/harmonicity; this assumption is arguably problematic, especially given recent claims that dyadic consonance is driven by periodicity/harmonicity, not interference (McDermott et al., 2010; Stolzenburg, 2015).

#### Bowling et al. (2018)

Bowling et al. (2018) primarily explain consonance in terms of periodicity/harmonicity, but also identify dissonance with chords containing pitches separated by less than 50 Hz. They argue that such intervals are uncommon in human vocalizations, and therefore elicit dissonance. We categorize this proposed effect under interference, in line with Parncutt et al.’s (2018) argument that these small intervals (in particular minor and major seconds) are strongly associated with interference.

### Interference: Pure Dyads

Pure-dyad interference models work by decomposing chords into their pure-tone components, and accumulating interference contributions from each pair of pure tones.

#### Plomp and Levelt (1965); Kameoka and Kuriyagawa (1969b)

Plomp and Levelt (1965) and Kameoka and Kuriyagawa (1969b) concurrently established an influential methodology for consonance modeling: Use perceptual experiments to characterize the consonance of pure-tone dyads, and estimate the dissonance of complex sonorities by summing contributions from each pure dyad. However, their original models are rarely used today, having been supplanted by later work.

#### Hutchinson and Knopoff (1978)

Hutchinson and Knopoff (1978) describe a pure-dyad interference model in the line of Plomp and Levelt (1965). Unlike Plomp and Levelt, Hutchinson and Knopoff sum dissonance contributions over all harmonics, rather than just neighboring harmonics. The original model is not fully algebraic, relying on a graphically depicted mapping between interval size and pure-dyad dissonance; a useful modification is the algebraic approximation introduced by Bigand, Parncutt, and Lerdahl (1996), which we adopt here (see also Mashinter, 2006).

Hutchinson and Knopoff (1978) only applied their model to complex-tone dyads. They later applied their model to complex-tone triads (Hutchinson & Knopoff, 1979), and for computational efficiency introduced an approximation decomposing the interference of a triad into the contributions of its constituent complex-tone dyads (see previous discussion of Huron, 1994). With modern computers, this approximation is unnecessary and hence rarely used.

#### Sethares (1993); Vassilakis (2001); Weisser and Lartillot (2013)

Several subsequent studies have preserved the general methodology of Hutchinson and Knopoff (1978) while introducing various technical changes. Sethares (1993) reformulated the equations linking pure-dyad consonance to interval size and pitch height. Vassilakis (2001) and Weisser and Lartillot (2013) subsequently modified Sethares’s (1993) model, reformulating the relationship between pure-dyad consonance and pure-tone amplitude. These modifications generally seem principled, but the resulting models have received little systematic validation.

#### Parncutt (1989); Parncutt and Strasburger (1994)

As discussed above (see the Periodicity/Harmonicity: Spectral Pattern Matching section), the pure tonalness measure of Parncutt (1989) and the pure sonorousness measure of Parncutt and Strasburger (1994) may be categorized as interference models. Unlike other pure-dyad interference models, these models address masking, not beating.

### Interference: Waveforms

Dyadic models present a rather simplified account of interference, and struggle to capture certain psychoacoustic phenomena such as effects of phase (e.g., Pressnitzer & McAdams, 1999) and waveform envelope shape (e.g., Vencovský, 2016) on roughness. The following models achieve a more detailed account of interference by modeling the waveform directly.

#### Leman (2000)

Leman’s (2000) synchronization index model measures beating energy within roughness-eliciting frequency ranges. The analysis begins with Immerseel and Martens’s (1992) model of the peripheral auditory system, which simulates the frequency response of the outer and middle ear, the frequency analysis of the cochlea, hair-cell transduction from mechanical vibrations to neural impulses, and transmission by the auditory nerve. Particularly important is the half-wave rectification that takes place in hair-cell transduction, which physically instantiates beating frequencies within the Fourier spectrum. Leman’s model then filters the neural transmissions according to their propensity to elicit roughness, and calculates the energy of the resulting spectrum as a roughness estimate. Leman illustrates model outputs for several amplitude-modulated tones, and for two-note chords synthesized with harmonic complex tones. The initial results seem promising, but we are unaware of any studies systematically fine-tuning or validating the model.

#### Skovenborg and Nielsen (2002)

Skovenborg and Nielsen’s (2002) model is conceptually similar to Leman’s (2000) model. The key differences are simulating the peripheral auditory system using the HUTear MATLAB toolbox (Härmä & Palomäki, 1999), rather than Immerseel and Martens’s (1992) model, and adopting different definitions of roughness-eliciting frequency ranges. The authors provide some illustrations of the model’s application to two-tone intervals of pure and complex tones. The model recovers some established perceptual phenomena, such as the dissonance elicited by small intervals, but also exhibits some undesirable behavior, such as multiple consonance peaks for pure-tone intervals, and oversensitivity to slight mistunings for complex-tone intervals. We are unaware of further work developing this model.

#### Aures (1985c); Daniel and Weber (1997); Wang et al. (2013)

Aures (1985c) describes a roughness model that has been successively developed by Daniel and Weber (1997) and Wang et al. (2013). Here we describe the model as implemented in Wang et al. (2013). Like Leman (2000) and Skovenborg and Nielsen (2002), the model begins by simulating the frequency response of the outer and middle ear, and the frequency analysis of the cochlea. Unlike Leman (2000) and Skovenborg and Nielsen (2002), the model does not simulate hair-cell transduction or transmission by the auditory nerve. Instead, the model comprises the following steps: (a) Extract the waveform envelope at each cochlear filter; (b) Filter the waveform envelopes to retain the beating frequencies most associated with roughness; (c) For each filter, compute the *modulation index*, summarizing beating magnitude as a proportion of the total signal; (d) Multiply each filter’s modulation index by a *phase impact factor*, capturing signal correlations between adjacent filters; high correlations yield higher roughness; (e) Multiply by a weighting factor identifying how different cochlear filters contribute more to the perception of roughness; (f) Square the result and sum over cochlear filters.

Unlike the models of Leman (2000) and Skovenborg and Nielsen (2002), these three models are presented alongside objective perceptual validations. However, these validations are generally restricted to relatively artificial and nonmusical stimuli.

#### Vencovský (2016)

Like Leman (2000); Skovenborg and Nielsen (2002), and Wang et al. (2013); Vencovský’s (2016) model begins with a sophisticated model of the peripheral auditory system. The model of Meddis (2011) is used for the outer ear, middle ear, inner hair cells, and auditory nerve; the model of Nobili, Vetešník, Turicchia, and Mammano (2003) is used for the basilar membrane and cochlear fluid. The output is a neuronal signal for each cochlear filter.

Roughness is then estimated from the neuronal signal’s envelope, or beating pattern. Previous models estimate roughness from the amplitude of the beating pattern; Vencovský’s (2016) model additionally accounts for the beating pattern’s shape. Consider a single oscillation of the beating pattern; according to Vencovský’s (2016) model, highest roughness is achieved when the difference between minimal and maximal amplitudes is large, and when the progression from minimal to maximal amplitudes (but not necessarily vice versa) is fast. Similar to previous models (Daniel & Weber, 1997; Wang et al., 2013), Vencovský’s (2016) model also normalizes roughness contributions by overall signal amplitudes, and decreases roughness when signals from adjacent cochlear channels are uncorrelated.

Vencovský (2016) validates the model on perceptual data from various types of artificial stimuli, including two-tone intervals of harmonic complex tones, and finds that the model performs fairly well. It is unclear how well the model generalizes to more complex musical stimuli.

### Culture

Cultural aspects of consonance perception have been emphasized by many researchers (see *Consonance Theories*), but we are only aware of one preexisting computational model instantiating these ideas: that of Johnson-Laird et al. (2012).

#### Johnson-Laird et al. (2012)

Johnson-Laird et al. (2012) provide a rule-based model of consonance perception in Western listeners. The model comprises three rules, organized in decreasing order of importance:

1 Chords consistent with a major scale are more consonant than chords only consistent with a minor scale, which are in turn more consonant than chords not consistent with either;
2 Chords are more consonant if they (a) contain a major triad and (b) all chord notes are consistent with a major scale containing that triad;
3 Chords are more consonant if they can be represented as a series of pitch classes each separated by intervals of a third, optionally including one interval of a fifth.

Unlike most other consonance models, this model does not return numeric scores, but instead ranks chords in order of their consonance. Ranking is achieved as follows: Apply the rules one at a time, in decreasing order of importance, and stop when a rule identifies one chord as more consonant than the other. This provides an estimate of cultural consonance.

Johnson-Laird et al. (2012) suggest that Western consonance perception depends both on culture and on roughness. They capture this idea with their *dual-process model*, which adds an extra rule to the cultural consonance algorithm, applied only when chords cannot be distinguished on the cultural consonance criteria. This rule predicts that chords are more consonant if they exhibit lower roughness. The authors operationalize roughness using the model of Hutchinson and Knopoff (1978).

The resulting model predicts chordal consonance rather effectively (Johnson-Laird et al., 2012; Stolzenburg, 2015). However, a problem with this model is that the rules are hand-coded on the basis of expert knowledge. The rules could represent cultural knowledge learned through exposure, but they could also explain post hoc rationalizations of perceptual phenomena. This motivates us to introduce an alternative corpus-based model, described below.

#### A corpus-based model of cultural familiarity

Here we introduce a simple corpus-based model of cultural familiarity, representing the hypothesis that listeners become familiar with chords in proportion to their frequency of occurrence in the listener’s musical culture, and that this familiarity positively influences consonance through the mere exposure effect (Zajonc, 2001). We simulate a Western listener’s musical exposure by tabulating the occurrences of different chord types in the Billboard dataset (Burgoyne, 2011), a large dataset of music from the U.S. charts. We reason that this dataset should provide a reasonable first approximation to the musical exposure of the average Western listener, but note that this approach could easily be tailored to the specific musical backgrounds of individual listeners. See the Method section for further details.

## Perceptual Analyses

Here we reanalyze consonance perception data from four previous studies (Bowling et al., 2018; Johnson-Laird et al., 2012; Lahdelma & Eerola, 2016; Schwartz et al., 2003). These datasets correspond to consonance judgments for Western musical chords as made by listeners from Western musical cultures. We focus in particular on the dataset from Bowling et al. (2018), as it contains considerably more chord types than previous datasets (see the Method section for details). We make all these datasets available in an accompanying R package, *inconData*.

Previous analyses of these datasets suffer from important limitations. Several studies show that a dataset is consistent with their proposed theory, but fail to test competing theories (Bowling et al., 2018; Schwartz et al., 2003). When competing theories are tested, each theory is typically operationalized using just one computational model (Johnson-Laird et al., 2012; Lahdelma & Eerola, 2016), and the choice of model is fairly arbitrary, because few comparative model evaluations are available in the literature. However, as we later show, models representing the same consonance theory can vary widely in performance. Furthermore, when multiple models are evaluated, parameter reliability is rarely considered, encouraging inferences to be made from statistically insignificant differences (Stolzenburg, 2015). Lastly, no studies simultaneously model contributions from periodicity/harmonicity, interference, and cultural familiarity, despite the implication from the empirical literature that all three phenomena may contribute to consonance perception.

Here we address these problems. Our primary goal is to reevaluate competing theories of consonance perception; our secondary goal is to facilitate future consonance research. Toward these goals, we compile 20 consonance models, 15 of which we implement in this paper’s accompanying R package, and five of which are available in publicly available audio analysis toolboxes (see Table 2). We systematically evaluate these 20 models on our perceptual data, providing future researchers an objective basis for model selection. We then assess the evidence for a composite theory of consonance perception, evaluating the extent to which periodicity/harmonicity, interference, and cultural familiarity simultaneously contribute to consonance judgments. We include the resulting composite consonance model in the incon package.[Table-anchor tbl2]

For practical reasons, we do not try to evaluate every model in the literature. In most cases, we only evaluate the latest published version of a given model, and avoid models with limited or discouraging perceptual validations (e.g., Leman, 2000; Skovenborg & Nielsen, 2002). We also omit one model on the grounds of its complexity (Vencovský, 2016). See the Method section for further details.

### Evaluating Models Individually

**Figure 2 fig2:**
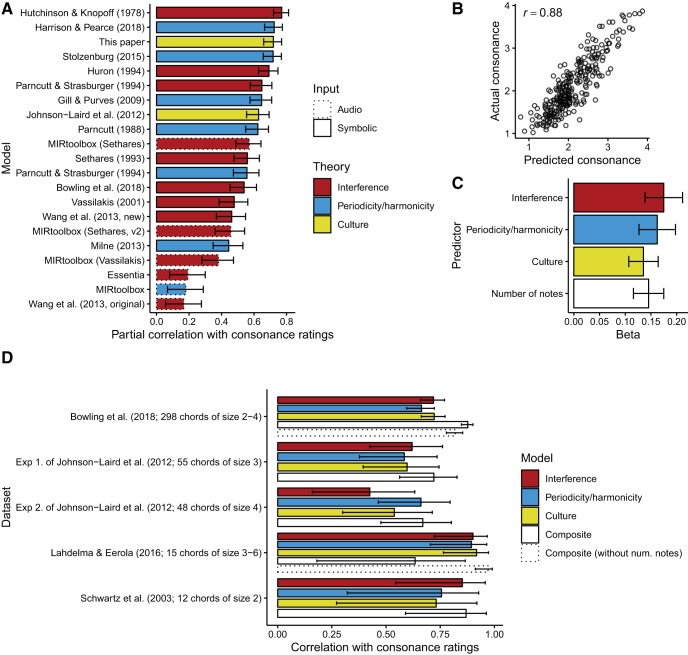
Results of the perceptual analyses. All error bars denote 95% confidence intervals. (A) Partial correlations between model outputs and average consonance ratings in the Bowling et al. (2018) dataset, after controlling for number of notes. (B) Predictions of the composite model for the Bowling et al. (2018) dataset. (C) Standardized regression coefficients for the composite model. (D) Evaluating the composite model across five datasets from four studies (Bowling et al., 2018; Johnson-Laird et al., 2012; Lahdelma & Eerola, 2016; Schwartz et al., 2003).

We begin by evaluating each consonance model individually on the Bowling et al. (2018) dataset (Figure 2A). Our performance metric is the partial correlation^15^ between model predictions and average consonance ratings, controlling for the number of notes in each chord, with the latter treated as a categorical variable. We control for number of notes to account for a design-related confound in Bowling et al. (2018) where stimulus presentation was blocked by the number of notes in each chord, potentially allowing participants to recalibrate their response scales for each new number of notes. We use predictive performance as an initial indicator of a model’s cognitive validity and practical utility.[Fig-anchor fig2]

#### Competing theories of consonance

The three best-performing models represent three different theories of consonance perception: interference (*r* = .77, 95% CI [.72, .81]), periodicity/harmonicity (*r* = .72, 95% CI [.66, .77]), and cultural familiarity (*r* = .72, 95% CI [.66, .77]). This similarity in performance is consistent with the idea that these three phenomena all contribute to consonance perception. Later we describe a regression analysis that provides a more principled test of this hypothesis.

#### Periodicity/harmonicity models

The most detailed periodicity/harmonicity model tested is that of Parncutt and Strasburger (1994), which incorporates various psychoacoustic phenomena including hearing thresholds, masking, and audibility saturation. However, this model’s performance (*r* = .56, 95% CI [.47, .63]) is matched or beaten by four periodicity/harmonicity models with essentially no psychoacoustic modeling (*r* = .62, .65, .72, .72). This suggests that these psychoacoustic details may be largely irrelevant to the relationship between periodicity/harmonicity and consonance.

#### Interference models

The interference models display an interesting trend in performance: Since Hutchinson and Knopoff (1978), performance has generally decreased, not increased. This is surprising, because each successive model typically incorporates a more detailed psychoacoustic understanding of the physics of amplitude fluctuation (exceptions are the complex-dyad models of Bowling et al., 2018, and Huron, 1994, and the masking model of Parncutt & Strasburger, 1994). This trend deserves to be explored further; an interesting possibility is that amplitude-fluctuation models fail to capture the potential contribution of masking to consonance (see the Consonance Theories section).

#### Cultural models

The new corpus-based consonance model (*r* = .72, 95% CI [.66, .77]) outperformed the rule-based consonance model (Johnson-Laird et al., 2012, *r* = .63, 95% CI [.55, .69]; 95% CI for the difference in correlations [.012, .017], after Zou, 2007).^16^

#### Symbolic versus audio models

Many of the algorithms evaluated here take symbolic inputs, reducing each stimulus to a few numbers representing its constituent pitches. The other algorithms take audio inputs, and therefore have access to the full spectral content of the stimulus. Given that consonance is sensitive to spectral content, one might expect the audio algorithms to outperform the symbolic algorithms. However, Figure 2A shows that this is not the case: Generally speaking, the symbolic algorithms outperformed the audio algorithms. Particularly bad results were seen for MIRtoolbox’s periodicity/harmonicity measure (*r* = .18, 95% CI [.07, .29]) and Essentia’s interference measure (*r* = .19, 95% CI [.08, .30]). Fairly good results were seen for MIRtoolbox’s interference measure, which performed best using its default settings (original Sethares model; *r* = .57, 95% CI [.49, .64]). Nonetheless, this model was still outperformed by several simple symbolic models (e.g., Huron, 1994; Parncutt, 1988).

#### Wang et al.’s (2013) Model

The original model of Wang et al. (2013) performed rather poorly (*r* = .17, 95% CI [.05, .28]). This poor performance was surprising, given the sophisticated nature of the model and its position in a well-established modeling tradition (Aures, 1985c; Daniel & Weber, 1997). Experimenting with the model, we found its performance to improve significantly upon disabling the “phase impact factors” component, whereby signal correlations between adjacent cochlear filters increase roughness (resulting partial correlation: *r* = .46, 95% CI [.37, .55]).

### A Composite Consonance Model

We constructed a linear regression model to test the hypothesis that multiple psychological mechanisms contribute to consonance perception. We fit this model to the Bowling et al. (2018) dataset, using four features representing interference, periodicity/harmonicity, cultural familiarity, and number of notes. The first three features corresponded to the three best-performing models in Figure 2A: Hutchinson and Knopoff’s (1978) roughness model, Harrison and Pearce’s (2018) harmonicity model, and the new cultural familiarity model. The fourth feature corresponded to the number of notes in the chord. All features were treated as continuous predictors.

The predictions of the resulting model are plotted in Figure 2B. The predictions correlate rather well with the ground truth (*r* = .88, 95% CI [.85, .90]), significantly outperforming the individual models in Figure 2A.

The resulting standardized regression coefficients are plotted in Figure 2C, with signs equated for ease of comparison. All four features contributed significantly and substantially to the model, each with broadly similar regression coefficients. As expected, interference was negatively related to consonance, whereas periodicity/harmonicity and cultural familiarity were positively related to consonance. Number of notes also contributed significantly, presumably reflecting participants recalibrating their response scales for blocks with different numbers of notes.

This pattern of regression coefficients supports our proposition that consonance is jointly determined by interference, periodicity/harmonicity, and cultural familiarity. Moreover, it implies that the effect of cultural familiarity on consonance perception is not solely mediated by learned preferences for periodicity/harmonicity (McDermott et al., 2010, 2016). However, the contribution of cultural familiarity should be taken with caution: It might alternatively reflect a noncultural contributor to consonance that is not captured by our periodicity/harmonicity or interference models, but that influences chord prevalences in music composition, and therefore correlates with our corpus-based cultural model. Future work could test this possibility by modeling individual differences in consonance perception as a function of the listener’s musical background.

### Generalizing to Different Datasets

A good predictive model of consonance should generalize outside the specific paradigm of Bowling et al. (2018). We therefore tested the new composite model on four additional datasets from the literature (Johnson-Laird et al., 2012; Lahdelma & Eerola, 2016; Schwartz et al., 2003). These datasets are relatively small, preventing model performance from being assessed with much reliability; nonetheless, they provide a useful initial test of the model’s generalizability. In each case, we assessed predictive performance by correlating model predictions with averaged consonance judgments for each stimulus, and benchmarked the composite model’s performance against that of its constituent submodels. For datasets varying the number of notes in each chord, we evaluated the composite model twice: once in its original form, and once removing the number of notes predictor, which we thought might be a design-related artifact from Bowling et al. (2018).

Johnson-Laird et al. (2012) provide two relevant datasets of consonance judgments, one for three-note chords (Experiment 1, 27 participants, 55 chords), and one for four-note chords (Experiment 2, 39 participants, 48 chords). Modeling these datasets, we found a trend for the composite model to outperform the individual submodels (Figure 2D). This trend is less clear in the second dataset, however, where interference performs particularly badly and periodicity/harmonicity performs particularly well, almost on a par with the composite model.^17^ A possible explanation is the fact that Johnson-Laird et al. (2012) purposefully undersampled chords containing adjacent semitones, thereby restricting the variation in interference.

Lahdelma and Eerola (2016) provide a dataset of consonance judgments from 410 participants for 15 chords in various transpositions, with the chords ranging in size from three to six notes. As transposition information was missing from the published dataset, we averaged consonance judgments over transpositions before computing the performance metrics. The composite model performed considerably worse (*r* = .63, 95% CI [.18, .87]) than the submodels (*r* > .89). This implied that the number-of-notes predictor was sabotaging predictions, and indeed, removing this predictor improved performance substantially (*r* = .97, 95% CI [.91, .99]). This pattern of results is consistent with the hypothesis that the number of notes effect observed in the Bowling et al. (2018) dataset was a design-related confound.

Schwartz et al. (2003) present data on the perceptual consonance of two-note chords as compiled from seven historic studies of consonance perception. The composite model performs well here (*r* = .87, 95% CI [.59, .96]), seemingly outperforming the submodels (.73 < *r* < .85), but the small dataset size limits the statistical power of these comparisons.

In a subsequent exploratory analysis, we benchmarked the composite model’s performance against the 10 best-performing models from Figure 2A. Model performance varied across datasets, and in some cases individual models achieved higher correlation coefficients than the composite model. However, no model significantly outperformed the composite model at a *p* < .05 level in any given dataset, even without correcting for multiple comparisons.

These evaluations provide qualified support for the composite model’s generalizability across datasets. Predictive performance is generally good, with the composite model typically matching or improving upon the performance of preexisting models. However, these inferences are constrained by the small dataset sizes of previous studies, which limit the precision of performance evaluations. A further limitation is that most previous studies do not manipulate the number of notes in the chord, which makes it difficult to test the generalizability of the number-of-notes effect observed in the Bowling et al. (2018) dataset. These limitations should be addressed in subsequent empirical work.

### Recommendations for Model Selection

Figure 2A shows that consonance models representing similar psychological theories can vary widely in performance. This highlights the danger of testing psychological theories with single computational models, especially when those models are relatively unvalidated. For example, Lahdelma and Eerola (2016) found that MIRtoolbox’s inharmonicity measure failed to predict consonance judgments, and concluded that periodicity/harmonicity does not contribute much to consonance. Our analyses replicate the low predictive power of MIRtoolbox’s inharmonicity measure (partial *r* < .2), but they show that other periodicity/harmonicity measures can predict consonance much better (partial *r* > .7). If Lahdelma and Eerola (2016) had selected a different periodicity/harmonicity model, their conclusions might therefore have been very different.

Figure 2A provides useful information for model selection. All else aside, models with higher predictive performance are likely to be better instantiations of their respective psychological theories. Here we selected the three best-performing models in Figure 2A, which usefully represent three different consonance theories: interference, periodicity/harmonicity, and cultural familiarity. However, several models reached similar levels of performance, and should be retained as good candidates for consonance modeling. Stolzenburg’s (2015) model performed especially well on the validation datasets, and should be considered a recommended alternative to Harrison and Pearce’s (2018) periodicity/harmonicity model. Likewise, if it is desirable for the model to be hand-computable, Huron’s (1994) model and Parncutt’s (1988) model both perform remarkably well given their simplicity. When only audio information is available, our results suggest that MIRtoolbox’s roughness measure is the best candidate for estimating consonance. In contrast, none of the audio-based periodicity/harmonicity measures were able to predict consonance.

There are some applications, such as emotion research, music information retrieval, or algorithmic music composition, where a composite model of consonance may be more useful than models representing individual consonance mechanisms. The composite model presented here would be well-suited for this role. However, the model would benefit from further tuning and validation, ideally on datasets varying chord spacing, tone spectra, and the number of notes in the chord.

## Corpus Analyses

We have argued that chord prevalences can provide a proxy for a listener’s musical exposure, and therefore can be used to model the contribution of cultural familiarity to consonance perception. However, these chord prevalences may themselves be partly determined by noncultural aspects of consonance perception, such as periodicity/harmonicity and interference.

A recent study by Parncutt et al. (2018) addressed these potential predictors of chord prevalences. The authors compiled a corpus of vocal polyphonic music spanning seven centuries of Western music, and correlated chord prevalences in this corpus with four features: interference, periodicity/harmonicity, diatonicity, and evenness. They predicted that interference and periodicity/harmonicity should respectively be negatively and positively related to chord prevalence, on account of these features’ respective contributions to perceptual consonance. They predicted that diatonic chords—chords played within the Western diatonic scale—should be more common, because the familiarity of the diatonic scale induces consonance in Western listeners. They also predicted that chord prevalences should be higher for chords whose notes are approximately evenly spaced, because even spacing is associated with efficient voice leading (Tymoczko, 2011).

Parncutt and colleagues tested these hypotheses by counting occurrences of 19 different three-note chord types in their dataset. They compiled a selection of formal models for each feature, and correlated model outputs with chord counts in their musical corpus, splitting the analysis by different musical periods. The observed correlations were generally consistent with the authors’ predictions, supporting the notion that perceptual consonance contributes to Western chord prevalences.

Although a useful contribution, this study has several important limitations. First, restricting consideration to just 19 chord types results in very imprecise parameter estimates. For example, a correlation coefficient of *r* = .5 has a 95% confidence interval ranging from .06 to .78; it is difficult to draw reliable inferences from such information. Second, pairwise correlations are unsuitable for quantifying causal effects when the outcome variable potentially depends on multiple predictor variables. Third, pairwise correlations can only capture linear relationships, and therefore cannot test more complex relationships between chord usage and consonance, such as the proposition that chord usage is biased toward intermediate levels of consonance (Lahdelma & Eerola, 2016). Fourth, the consonance models are simple note-counting models, which often lack specificity to the feature being analyzed. For example, interference is modeled using the dyadic consonance model of Huron (1994), but this model is built on dyadic consonance judgments which have recently been attributed to periodicity/harmonicity, not interference (McDermott et al., 2010; Stolzenburg, 2015).

Here we address these limitations, analyzing chord occurrences in three large corpora spanning the last thousand years of Western music: a corpus of classical scores (Viro, 2011), a corpus of jazz lead sheets (Broze & Shanahan, 2013), and a corpus of harmonic transcriptions of popular songs (Burgoyne, 2011). Instead of restricting consideration to 19 chord types, we tabulated prevalences for all 2,048 possible pitch-class chord types (see the Method section for further details). Instead of pairwise correlations, we constructed polynomial regression models capable of capturing nonlinear effects of multiple simultaneous predictors. Instead of simple note-counting models, we used the best-performing consonance models from Figure 2A: Hutchinson and Knopoff’s (1978) interference model, and Harrison and Pearce’s (2018) periodicity/harmonicity model.

We were particularly interested in how interference and periodicity/harmonicity contributed to chord prevalence. However, we also controlled for the number of notes in the chord, reasoning that this feature is likely to have constrained chord usage on account of practical constraints (e.g., the number of instruments in an ensemble).

Analyzing interference and periodicity/harmonicity allows us to revisit recent claims that consonance is primarily determined by periodicity/harmonicity and not interference (Cousineau et al., 2012; McDermott et al., 2010, 2016). If consonance is indeed predicted primarily by periodicity/harmonicity, we would expect periodicity/harmonicity to be an important predictor of Western chord prevalences, and that interference should have little predictive power after controlling for periodicity/harmonicity. Conversely, if consonance derives from both interference and periodicity/harmonicity, then we might expect both features to contribute to chord prevalences.

Compiling chord prevalences requires a decision about how to categorize chords into chord types. Here we represented each chord as a *pitch-class chord type*, defined as a pitch-class set expressed relative to the bass pitch class. This representation captures the perceptual principles of octave invariance (the chord type is unchanged when chord pitches are transposed by octaves, as long as they do not move below the bass note) and transposition invariance (the chord type is unchanged when all the chord’s pitches are transposed by the same interval).

Hutchinson and Knopoff’s model requires knowledge of precise pitch heights, which are not available in pitch-class chord type representations. We therefore assigned pitch heights to each chord type by applying the automatic chord voicing algorithm of Harrison and Pearce (2019; see the Method section for details).

Chord type prevalences could be operationalized in various ways. Ideally, one might sum the temporal duration of each chord type over all of its occurrences, perhaps weighting compositions by their popularity to achieve the best representation of a given musical style. However, chord durations and composition popularity were not available for our classical and jazz datasets. We therefore operationalized chord type prevalences as the total number of occurrences of each chord type, excluding immediate repetitions of the same chord (see the Method section).

We constructed three orthogonal polynomial regression models predicting log-transformed chord counts from interference, periodicity/harmonicity, and number of notes. The classical, jazz, and popular corpora contributed 2,048, 118, and 157 data points respectively, corresponding to the unique chord types observed in each corpus and their respective counts. Each corpus was assigned its own polynomial order by minimizing the Bayesian Information Criterion for the fitted model; the classical, jazz, and popular datasets were thereby assigned third-order, first-order, and second-order polynomials respectively.

**Figure 3 fig3:**
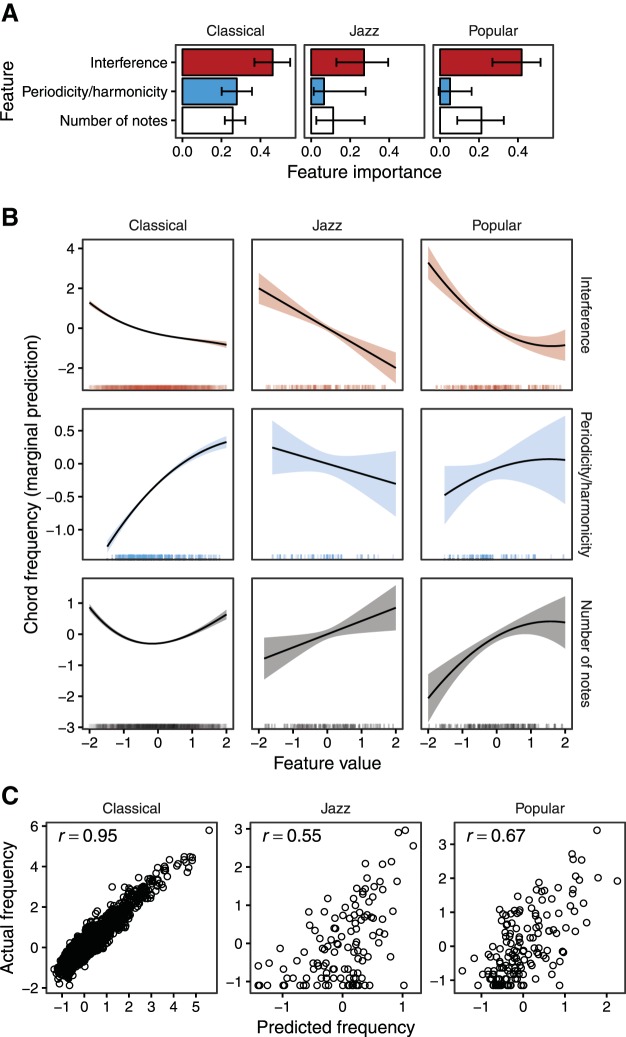
Results of the corpus analyses. (A) Feature importance as assessed by model reliance (Fisher et al., 2018), with error bars indicating 95% confidence intervals (bias-corrected and accelerated bootstrap, 100,000 replicates, DiCiccio & Efron, 1996). (B) Marginal effects of each feature, calculated using *z*-scores for feature values and for chord frequencies. The shaded areas describe 95% confidence intervals, and distributions of feature observations are plotted at the bottom of each panel. Distributions for the “number of notes” feature are smoothed to avoid overplotting. (C) Predicted and actual chord-type frequencies, alongside corresponding Pearson correlation coefficients.

Figure 3A quantifies each predictor’s importance using *model reliance* (Fisher, Rudin, & Dominici, 2018, see the Method section for details). Across the three genres, interference was consistently the most important predictor, explaining c. 20% to 50% of the variance in chord prevalences. Periodicity/harmonicity was also an important predictor for classical music, but not for popular or jazz music. Number of notes predicted chord prevalences in all three genres, explaining about half as much variance as interference.[Fig-anchor fig3]

Figure 3B plots the marginal effects of each predictor, showing how feature values map to predictions. Interference had a clear negative effect on chord prevalence in all three genres, consistent with the notion that interference evokes dissonance, causing it to be disliked by listeners and avoided by composers. Periodicity/harmonicity had a clear positive effect on chord prevalence in the classical dataset, consistent with the idea that periodicity/harmonicity evokes consonance and is therefore promoted by composers (Figure 3B). The effect of periodicity/harmonicity was less strong in the popular and jazz datasets, taking the form of a weak positive effect in the popular dataset and a weak negative effect in the jazz dataset.

Figure 3C summarizes the predictive performances of the three regression models. Generally speaking, predictive performances were high, indicating that consonance and number of notes together explain a large part of Western chord prevalences. However, the strength of this relationship varied by musical style, with the classical dataset exhibiting the strongest relationship and the jazz dataset the weakest relationship.

In sum, these results weigh against the claim that consonance is primarily determined by periodicity/harmonicity and not interference (Bowling & Purves, 2015; Bowling et al., 2018; McDermott et al., 2010). Across musical genres, interference seems to have a strong and reliable negative effect on chord prevalences. Periodicity/harmonicity also seems to influence chord prevalences, but its effect is generally less strong, and the nature of its contribution seems to vary across musical genres.

## Discussion

Recent research argues that consonance perception is driven not by interference but by periodicity/harmonicity, with cultural differences in consonance perception being driven by learned preferences for the latter (Cousineau et al., 2012; McDermott et al., 2010, 2016). We reassessed this claim by reviewing a wide range of historic literature, modeling perceptual data from four previous empirical studies, and conducting corpus analyses spanning a thousand years of Western music composition. We concluded that interference contributes significantly to consonance perception in Western listeners, and that cultural aspects of consonance perception extend past learned preferences for periodicity/harmonicity. Instead, consonance perception in Western listeners seems to be jointly determined by interference, periodicity/harmonicity perception, and learned familiarity with particular musical sonorities.

This multicomponent account of consonance is broadly consistent with several previous claims in the literature. Terhardt (1974, 1984) has emphasized the role of roughness and harmonicity in determining consonance, and Parncutt and colleagues have argued that consonance depends on roughness, harmonicity, and familiarity (Parncutt & Hair, 2011; Parncutt et al., 2018). Scientific preferences for parsimony may have caused these multicomponent accounts to be neglected in favor of single-component accounts, but our analyses demonstrate the necessity of the multicomponent approach.

This consolidation of multiple psychological mechanisms makes an interesting parallel with historic pitch perception research, where researchers strove to demonstrate whether pitch perception was driven by place coding or temporal coding (see de Cheveigné, 2005 for a review). It proved difficult to falsify either place coding or temporal coding theories, and many researchers now believe that both mechanisms play a role in pitch perception (e.g., Bendor, Osmanski, & Wang, 2012; Moore & Ernst, 2012).

Like most existing consonance research, our analyses were limited to Western listeners and composers, and therefore we can only claim to have characterized consonance in Westerners. Previous research has identified significant cross-cultural variation in consonance perception (Florian, 1981; Maher, 1976; McDermott et al., 2016); we suggest that this cross-cultural variation might be approximated by varying the regression coefficients in our composite consonance model. For example, listeners familiar with beat diaphony seem to perceive interference as consonant, not dissonant (Florian, 1981); this would be reflected in a reversed regression coefficient for interference. While the regression coefficients might vary cross-culturally, it seems plausible that the model’s underlying predictors—interference, periodicity/harmonicity, familiarity—might recur cross-culturally, given the cross-cultural perceptual salience of these features (McDermott et al., 2016).

Our conclusions are not inconsistent with vocal-similarity theories of consonance perception (Bowling & Purves, 2015; Bowling et al., 2018; Schwartz et al., 2003). According to these theories, certain chords sound consonant because they particularly resemble human vocalizations. These theories usually emphasize periodicity/harmonicity as a salient feature of human vocalizations, but they could also implicate interference as a feature avoided in typical vocalizations (Bowling et al., 2018) but used to convey distress in screams (Arnal, Flinker, Kleinschmidt, Giraud, & Poeppel, 2015). It seems plausible that these mechanisms contribute a universal bias to perceive periodicity/harmonicity as pleasant and interference as unpleasant. Nonetheless, these biases must be subtle enough to allow cultural variation, if we are to account for musical cultures that lack preferences for periodicity/harmonicity (McDermott et al., 2016) or that consider interference to be pleasant (Florian, 1981).

Our analyses were limited by the computational models tested. It would be interesting to develop existing models further, perhaps producing a version of Bowling et al.’s (2018) periodicity/harmonicity model that accepts arbitrary tunings, or a version of Parncutt and Strasburger’s (1994) model without discrete-pitch approximations. It would also be interesting to test certain models not evaluated here, such as Boersma’s (1993) model and Vencovský’s (2016) model.

Our perceptual analyses were limited by the available empirical data. Future work should expand these datasets, with particular emphasis on varying voicing, tone spectra, and number of notes in the chord. Such datasets would be essential for testing the generalizability of our models.

Our perceptual analyses marginalized over participants, producing an average consonance rating for each chord. This approach neglects individual differences, which can provide an important complementary perspective on consonance perception (McDermott et al., 2010). When suitable empirical datasets become available, it would be interesting to investigate how the regression weights in Figure 2C vary between participants.

Our corpus analyses presented very broad approximations to musical genres, aggregating over a variety of musical styles and time periods. It would be interesting to apply these methods to more specific musical styles, or indeed to individual composers. It would also be interesting to investigate the evolution of consonance treatment over time. As we analyze music compositions dating further back in history, we should expect the chord distributions to reflect consonance perception in historic listeners rather than modern listeners. Such analyses could potentially shed light on how consonance perception has changed over time (Parncutt et al., 2018).

Our three corpora were constructed in somewhat different ways. The classical corpus was derived from published musical scores; the jazz corpus constitutes a collection of lead sheets; the popular corpus comprises expert transcriptions of audio recordings. This heterogeneity is both an advantage, in that it tests the generalizability of our findings to different transcription techniques, and a disadvantage, in that it reduces the validity of cross-genre comparisons. Future work could benefit from corpora with both stylistic diversity and consistent construction.

We hope that our work will facilitate future psychological research into consonance. Our incon package makes it easy to test diverse consonance models on new datasets, and it can be easily extended to add new models. Our inconData package compiles the perceptual datasets analyzed here, making it easy to test new consonance models on a variety of perceptual data.

This work should also have useful applications in computational musicology and music information retrieval. Our composite consonance model provides a principled way to operationalize the net consonance of a musical chord, while our model evaluations provide a principled way to operationalize individual consonance theories. Our software provides a consistent and easy-to-use interface to these models, facilitating their application to new datasets.

## Method

### Models

The models evaluated in this paper are available from three software sources: the incon package, MIRtoolbox,^18^ and Essentia.^19^ Unless otherwise mentioned, all incon models represent unaltered versions of their original algorithms as described in the cited literature, with the exception that all idealized harmonic spectra comprised exactly 11 harmonics (including the fundamental frequency), with the *i*th harmonic having an amplitude of *i*^−1^, and assuming incoherence between tones for the purpose of amplitude summation. We clarify some further details below.

#### Harrison and Pearce (2018); Milne (2013)

These algorithms have three free parameters: the number of harmonics modeled in each complex tone, the harmonic roll-off rate (ρ), and the standard deviation of the Gaussian smoothing distribution (σ). We set the number of harmonics to 11 (including the fundamental frequency), and set the other two parameters to the optimized values in Milne and Holland (2016): a roll-off of ρ = 0.75, and a standard deviation of σ = 6.83 cents.

#### Hutchinson and Knopoff (1978)

Our implementation is based on Mashinter (2006), whose description includes a parametric approximation for the relationship between interval size and pure-dyad dissonance (see also Bigand et al., 1996).

#### Sethares (1993)

Our implementation is primarily based on Sethares (1993), but we include a modification suggested in later work (Sethares, 2005; Weisser & Lartillot, 2013) where pure-dyad consonance is weighted by the minimum amplitude of each pair of partials, not the product of their amplitudes.

#### Wang et al. (2013)

Our implementation of Wang et al.’s (2013) algorithm takes symbolic input and expresses each input tone as an idealized harmonic series. Time-domain analyses are conducted with a signal length of 1 s and a sample rate of 44,000. Frequency-domain analyses are conducted in the range 1–44,000 Hz with a resolution of 1 Hz. An interactive demonstration of the algorithm is available at http://shiny.pmcharrison.com/wang13.

#### Essentia: Interference

We used Version 2.1 of Essentia. We analyzed each audio file using the “essentia_streaming_extractor_music” feature extractor, and retained the mean estimated dissonance for each file.

#### MIRtoolbox: Interference

We used Version 1.6.1 of MIRtoolbox, and computed roughness using the “mirroughness” function. The function was applied to a single window spanning the entire length of the stimulus.

We evaluated this model in several configurations (see Figure 2A):

1 “Sethares” denotes the default model configuration, which implements the dissonance model of Sethares (2005), but with pure-tone dyad contributions being weighted by the *product* of their amplitudes (see Sethares, 1993);
2 “Sethares, v2” denotes the “Min” option in MIRtoolbox, where pure-tone dyad contributions are weighted by the *minimum* of their amplitudes, after Weisser and Lartillot (2013) (see also Sethares, 2005);
3 “Vassilakis” denotes MIRtoolbox’s implementation of Vassilakis’s (2001) model.

#### Johnson-Laird et al. (2012)

Johnson-Laird et al.’s (2012) algorithm may be separated into a cultural and an interference component, with the latter corresponding to Hutchinson and Knopoff’s (1978) model. The cultural model assigns each chord to a consonance category, where categories are ordered from consonant to dissonant, and chords within a category are considered to be equally consonant. In our implementation, these consonance categories are mapped to positive integers, such that higher integers correspond to greater dissonance. These integers constitute the algorithm’s outputs.

#### Corpus-based model of cultural familiarity

This model estimates a listener’s unfamiliarity with a given chord type from its rarity in a musical corpus. Here we use the Billboard dataset (Burgoyne, 2011), a corpus of popular songs sampled from the Billboard magazine’s “Hot 100” chart in the period 1958–1991. This corpus is used as a first approximation to an average Western listener’s prior musical exposure. We represent each chord in this corpus as a *pitch-class chord type*, defined as the chord’s pitch-class set expressed relative to the chord’s bass note. For example, a chord with MIDI note numbers {66, 69, 74} has a pitch-class chord type of {0, 3, 8}. We count how many times each of the 2,048 possible pitch-class chord types occurs in the corpus, and add 1 to the final count. Unfamiliarity is then estimated as the negative natural logarithm of the chord type’s count.

#### Composite model

The composite model’s unstandardized regression coefficients are provided to full precision in Table 3. Consonance is estimated by computing the four features listed in Table 3, multiplying them by their respective coefficients, and adding them to the intercept coefficient. Number of notes corresponds to the number of distinct pitch classes in the chord; interference is computed using Hutchinson and Knopoff’s (1978) model; periodicity/harmonicity is computed using Harrison and Pearce’s (2018) model; culture corresponds to the new corpus-based cultural model.[Table-anchor tbl3]

It is unclear whether the effect of number of notes generalizes outside the dataset of Bowling et al. (2018) (see the Perceptual Analyses section). We therefore recommend setting the number of notes coefficient to zero when applying the model to new datasets.

### Software

We release two top-level R packages along with this paper. The first, incon, implements the symbolic consonance models evaluated in this paper (see Table 2).^20^ The second, inconData, compiles the perceptual datasets that we analyzed.^21^ Tutorials are available alongside these packages.

The incon package depends on several low-level R packages that we also release along with this paper, namely *bowl18*, *corpdiss*, *dycon*, *har18*, *hcorp*, *hrep*, *jl12*, *parn88*, *parn94*, *stolz15*, and *wang13*. These packages provide detailed interfaces to individual consonance models and tools for manipulating harmony representations.

Our software, analyses, and article were all created using the programming language R (R Core Team, 2017), and benefited in particular from the following open-source packages: *bookdown*, *boot*, *checkmate*, *cocor*, *cowplot*, *dplyr*, *ggplot2*, *glue*, *gtools*, *hht*, *knitr*, *jsonlite*, *magrittr*, *margins*, *memoise*, *numbers*, *papaja*, *phonTools*, *plyr*, *purrr*, *Rdpack*, *readr*, *rmarkdown*, *testthat*, *tibble*, *tidyr*, *usethis*, *withr*, and *zeallot*. Our analysis code is freely available online.^22^

### Perceptual Datasets

The following datasets are all included in our inconData Package.

#### Bowling et al. (2018)

This study collected consonance judgments for all possible 12 two-note chord types, 66 three-note chord types, and 220 four-note chord types that can be formed from the Western chromatic scale within a one-octave span of the bass note.^23^ An advantage of this dataset is its systematic exploration of the chromatic scale; a disadvantage is its restricted range of voicings.

Each chord tone was pitched as a just-tuned interval from the bass note.^24^ This approach was presumably chosen because Bowling et al.’s (2018) periodicity/harmonicity model requires just tuning, but it should be noted that just tuning itself is not commonly adopted in Western music performance (e.g., Karrick, 1998; Kopiez, 2003; Loosen, 1993). It should also be noted that tuning a chord in this way does not ensure that the intervals between nonbass notes are just-tuned, and certain chords can sound unusually dissonant as a result compared with their equal-tempered equivalents.

Each chord type was assigned a bass note such that the chord’s mean fundamental frequency would be equal to middle C, approximately 262 Hz. The resulting chords were played using the “Bosendorfer Studio Model” synthesized piano in the software package “Logic Pro 9.”

The participant group numbered 30 individuals. Of these, 15 were students at a Singapore music conservatory, each having taken weekly formal lessons in Western tonal music for an average of 13 years (*SD* = 3.8). The remaining 15 participants were recruited from the University of Vienna, and averaged less than a year of weekly music lessons prior to the study (*SD* = 1.1).

Participants were played single chords, and asked to rate consonance on a 4-point scale, where consonance was defined as “the musical pleasantness or attractiveness of a sound.” Participants were free to listen to the same chord multiple times before giving a rating. Stimulus presentation was blocked by the number of notes in each chord, with stimulus presentation randomized within blocks. This presents an unfortunate potential confound; if consonance differed systematically across chords containing different numbers of notes, this may have caused participants to recalibrate their scale usage across blocks.

#### Johnson-Laird et al. (2012), Experiment 1

This experiment collected consonance ratings for all 55 possible three-note *pitch-class chord types*, where a pitch-class chord type is defined as a chord’s pitch-class set expressed relative to the bass pitch class. These chords were voiced so that each chord spanned approximately 1.5 octaves. All chords were played with synthesized piano using the “Sibelius” software package.

The participant group numbered 27 individuals from the Princeton University community. Some were nonmusicians, some were musicians, but all were familiar with Western music.

Participants were played single chords, and asked to rate dissonance on a seven-point scale, where dissonance was defined as “unpleasantness.” Each chord was only played once, with presentation order randomized across participants.

#### Johnson-Laird et al. (2012), Experiment 2

This experiment collected consonance ratings for 43 four-note pitch-class chord types. The rationale for chord selection is detailed in Johnson-Laird et al. (2012); particularly relevant is the decision to undersample chords containing three adjacent semitones, which may have mitigated contributions of interference to their results.

The participant group numbered 39 individuals from the Princeton University community. All other aspects of the design were equivalent to Experiment 1.

#### Lahdelma and Eerola (2016)

This experiment collected consonance ratings for 15 different *pitch chord types*, where a pitch chord type is defined as a chord’s pitch set expressed relative to its bass pitch. These chords ranged in size from three to six notes. The full rationale for chord selection is detailed in Lahdelma and Eerola (2016), but the main principle was to select chords with high consonance according to Huron’s (1994) dyadic consonance model, and with varying levels of cultural familiarity according to Tymoczko (2011). Because Huron’s model primarily captures interference (see the Computational Models section), this approach is likely to minimize between-stimulus variation in interference, potentially reducing the predictive power of interference models within this dataset. All chords were played using the synthesized “Steinway D Concert Grand” piano in the software package “Ableton Live 9” with the “Synthogy Ivory Grand Pianos II” plug-in.

The participant group was tested online, and numbered 418 individuals after quality-checking. These participants represented 42 different nationalities, with 91.7% coming from Europe and the Americas.

Each participant was played 30 stimuli comprising the 15 chord types each at a “low” and a “high” transposition, with the precise transpositions of these chord types randomly varying within an octave for each transposition category. Unfortunately, precise transposition information seems not to be preserved in the published response data. For the purpose of estimating interference, we therefore represented each chord type with a bass note of G4 (c. 392 Hz), corresponding to the middle of the range of bass notes used in the original study.

Participants were instructed to rate each chord on five 5-point scales; here we restrict consideration to the “consonance” scale. Curiously, “consonance” was defined as “How smooth do you think the chord is,” with the scale’s extremes being termed “rough” and “smooth.” This definition resembles more a definition of roughness than consonance, a potential problem for interpreting the study’s results.

#### Schwartz et al. (2003)

This dataset provides consonance ratings for the 12 two-note chord types in the octave, aggregated over seven historic studies. Each study produced a rank ordering of these two-note chords; these rank orderings were then summarized by taking the median rank for each chord.

### Musical Corpora

#### Classical scores

The classical dataset was derived from the Peachnote music corpus (Viro, 2011).^25^ This corpus compiles more than 100,000 scores from the Petrucci Music Library (IMSLP, http://imslp.org), spanning several hundred years of Western art music (1198–2011). Each score was digitized using optical music recognition software. In the resulting dataset, each datum represents a distinct “vertical slice” of the score, with new slices occurring at new note onsets, and including sustained notes sounded at previous onsets. We preprocessed this dataset to a pitch-class chord-type representation, where each chord is represented as a pitch-class set expressed relative to its bass pitch class. The resulting dataset numbered 128,357,118 chords.

#### Jazz lead sheets

The jazz dataset was derived from the iRb corpus (Broze & Shanahan, 2013). The iRb corpus numbers 1,186 lead sheets for jazz compositions, where each lead sheet specifies the underlying chord sequence for a given composition. These lead sheets were compiled from an online forum for jazz musicians. In the original dataset, chords are represented as textual tokens, such as “C7b9”; we translated all such tokens into a prototypical pitch-class chord-type representation, such as {0, 1, 4, 7, 10}. This process misses the improvisatory chord alterations that typically happen during jazz performances, but nonetheless should provide a reasonable first approximation to the performed music. Chord counts were only incremented on chord changes, not chord repetitions; section repeats were omitted. The resulting dataset numbered 42,822 chords.

#### Popular transcriptions

The popular dataset was derived from the McGill Billboard corpus (Burgoyne, 2011), which comprised chord sequences for 739 unique songs as transcribed by expert musicians. As with the iRb dataset, we translated all chord tokens into prototypical pitch-class chord-type representations, omitting section repeats, and only incrementing chord counts on each chord change. The resulting dataset numbered 74,093 chords.

### Corpus Analyses

We transformed each of our corpora to *pitch-class chord type* representations, where each chord is represented as a pitch-class set relative to the chord’s bass note. We then counted occurrences of pitch-class chord types in our three corpora.

For the purpose of applying Hutchinson and Knopoff’s (1978) interference model, we assigned pitch heights to each chord type using the automatic chord voicing algorithm of Harrison and Pearce (2019). This model was originally designed for voicing chord sequences, but it can also be applied to individual chords. Its purpose is to find an idiomatic assignment of pitch heights to pitch classes that reflects the kind of psychoacoustic considerations implicitly followed by traditional Western composers (e.g., Huron, 2001). As applied here, the model minimized the following linear combination of features:

$$\begin{array}{ll} 8.653 & \times \;\text{interference}\\ & +\;1.321\;\times \;|\;5-\text{number}\;\text{of}\;\text{notes}\;|\\ & +\;0.128\;\times \;|\;60-\text{mean}\;\text{pitch}\;\text{height}\;| \end{array}$$ 2

where “interference” refers to the raw output of Hutchinson and Knopoff’s model, “number of notes” refers to the number of unique pitches in the chord voicing, and “mean pitch height” corresponds to the mean of the chord’s pitches as expressed in MIDI note numbers.^26^ In other words, the model minimized the chord’s interference while preferring chords containing (close to) five discrete pitches with a mean pitch height close to middle C (c. 262 Hz). These model parameters correspond to the optimal parameters that Harrison and Pearce (2019) derived from a dataset of 370 chorale harmonizations by J. S. Bach, but with the target number of notes changed from four to five. Chord voicings were restricted to the two octaves surrounding middle C, and were permitted to contain no more than five notes or the number of pitch classes in the chord type, whichever was greater.

We used polynomial regression to capture nonlinear relationships between chord features and chord prevalences. We used orthogonal polynomials, as computed by the R function “poly,” to avoid numerical instability, and we used the R package “margins” to compute marginal predictions for the resulting models.

Standardized regression coefficients become harder to interpret as the polynomial degree increases. We instead assessed feature importance using *model reliance* (Fisher et al., 2018), a permutation-based metric commonly used for assessing feature importance in random forest models (Breiman, 2001). Model reliance may calculated by computing two values: the model’s original predictive accuracy, and the model’s predictive accuracy after randomly permuting the feature of interest (without refitting the model). Model reliance is then defined as the difference in these accuracies: The greater the difference, the more the model relies on the feature of interest. Here we used *R*^2^ as the performance metric, and computed confidence intervals for our model reliance estimates using bias-corrected accelerated bootstrapping with 100,000 replicates (DiCiccio & Efron, 1996).

## Figures and Tables

**Table 1 tbl1:** Summarized Evidence for the Mechanisms Underlying Western Consonance Perception

Evidence	Interference	Periodicity/harmonicity	Culture
Stimulus effects			
Tone spectra	✓		
Pitch height	✓		
Dichotic presentation	✠		
Familiarity			(✓)
Chord structure	(✓)	(✓)	(✓)
→*This paper: Perceptual analyses*	✓	✓	(✓)
Listener effects			
Western listeners	(✗)	✓	
Congenital amusia		✓	
Non-Western listeners			✓
Infants			(✠)
Animals			(✠)
Composition effects			
Musical scales	✓		
Manipulation of interference	✓		✓
Chord spacing (Western music)	✓		
Chord prevalences (Western music)	(✓)	(✓)	
→*This paper: Corpus analyses*	✓	✓	
*Note*. Each row identifies a section in *Current Evidence*. “✓” denotes evidence that a mechanism contributes to Western consonance perception. “✗” denotes evidence that a mechanism is *not* relevant to Western consonance perception. “✠” denotes evidence that a mechanism is insufficient to explain Western consonance perception. Parentheses indicate tentative evidence; blank spaces indicate a lack of evidence.			

**Table 2 tbl2:** Consonance Models Evaluated in the Present Work

Reference	Original name	Input	Implementation
Periodicity/harmonicity			
Gill and Purves (2009)	Percentage similarity	Symbolic	incon (bowl18)
Harrison and Pearce (2018)	Harmonicity	Symbolic	incon (har18)
Milne (2013)	Harmonicity	Symbolic	incon (har18)
Parncutt (1988)	Root ambiguity	Symbolic	incon (parn88)
Parncutt and Strasburger (1994)	Complex sonorousness	Symbolic	incon (parn94)
Stolzenburg (2015)	Smoothed relative periodicity	Symbolic	incon (stolz15)
Lartillot, Toiviainen, and Eerola (2008)	Inharmonicity	Audio	MIRtoolbox
Interference			
Bowling, Purves, and Gill (2018)	Absolute frequency intervals	Symbolic	incon (bowl18)
Huron (1994)	Aggregate dyadic consonance	Symbolic	incon
Hutchinson and Knopoff (1978)	Dissonance	Symbolic	incon (dycon)
Parncutt and Strasburger (1994)	Pure sonorousness	Symbolic	incon (parn94)
Sethares (1993)	Dissonance	Symbolic	incon (dycon)
Vassilakis (2001)	Roughness	Symbolic	incon (dycon)
Wang, Shen, Guo, Tang, and Hamade (2013)	Roughness	Symbolic	incon (wang13)
Bogdanov et al. (2013)	Dissonance	Audio	Essentia
Lartillot, Toiviainen, and Eerola (2008)	Roughness (after Sethares)	Audio	MIRtoolbox
Lartillot, Toiviainen, and Eerola (2008)	Roughness (after Vassilakis)	Audio	MIRtoolbox
Weisser and Lartillot (2013)	Roughess (after Sethares)	Audio	MIRtoolbox
Culture			
Johnson-Laird, Kang, and Leong (2012)	Tonal dissonance	Symbolic	incon (jl12)
This paper	Corpus dissonance	Symbolic	incon (corpdiss)
*Note*. “Reference” identifies the literature where the model or relevant software package was originally presented. “Original name” corresponds to the name of the model (or corresponding psychological feature) in the reference literature. “Input” describes the input format for the model implementations used in this paper. “Implementation” describes the software used for each model implementation, with “incon” referring to the incon package that accompanies this paper, and “Essentia” and “MIRtoolbox” corresponding to the software presented in Bogdanov et al. (2013) and Lartillot et al. (2008) respectively. Terms in parentheses identify the low-level R packages that underpin the incon package, and that provide extended access to individual models.			

**Table 3 tbl3:** Unstandardized Regression Coefficients for the Composite Consonance Model

Term	Coefficient
Intercept	0.628434666589357
Number of notes	0.422267698605598
Interference	−1.62001025973261
Periodicity/harmonicity	1.77992362857478
Culture	−0.0892234643584134
*Note*. These regression coefficients are presented to full precision for the sake of exact reproducibility, but it would also be reasonable to round the coefficients to c. 3 significant figures. When generalizing outside the dataset of Bowling et al. (2018), we recommend setting the number of notes coefficient to zero.	

## Footnotes

1 By “Western music” we refer broadly to the musical traditions of Europe and music derived from these traditions; by “Western listeners” we refer to listeners from these musical traditions.

2 Note that in practice, however, the octave is often stretched slightly beyond a 2:1 ratio (e.g. Rakowski, 1990).

3 In particular, the fundamental frequency is equal to the greatest common divisor of the frequency components.

4 Periodicity theories of consonance predating the 20th century can be found in the work of Galileo Galilei, Gottfried Wilhelm Liebniz, Leonhard Euler, Theodor Lipps, and A. J. Polak (Plomp & Levelt, 1965).

5 Earlier work in a similar line can be found in Sorge (1747), cited in Plomp and Levelt (1965) and Sethares (2005).

6 Audio examples from Sethares (2005) are available at http://sethares.engr.wisc.edu/html/soundexamples.html.

7 For example, Stolzenburg (2015, Table 4) tabulates correlation coefficients for 15 consonance models as evaluated on 12 dyads; the median correlation of .939 has a 95% confidence interval spanning from .79 to .98, encompassing all but one of the reported coefficients.

8 Zero of twelve of their consonant chords contain intervals smaller than a minor third, whereas 15/16 of their dissonant chords contain such intervals.

9 Toro and Crespo-Bojorque (2017) also claim that consonance discrimination has been demonstrated in black-capped chickadees, but we disagree in their interpretation of the cited evidence (Hoeschele, Cook, Guillette, Brooks, & Sturdy, 2012).

10 It would be worth testing this formally, applying periodicity/harmonicity consonance models (e.g. Harrison & Pearce, 2018) to the inharmonic tone spectra of Gamelan and Thai classical music, and relating the results to scale structure.

11 For example, a correlation coefficient of *r* = 0.5 with 19 triads has a 95% confidence interval of [0.06, 0.78].

12 A chord is just-tuned when its pitches are drawn from a just-tuned scale. A just-tuned scale is a scale tuned to maximize ratio simplicity.

13 The weights assigned to each harmonic differ between studies; Terhardt (1982) used binary weights, but Parncutt (1988) introduced graduated weights, which he updated in later work (see Parncutt, 2006a).

14 These measures were later termed pure and complex *sonorousness* by Parncutt and Strasburger (1994).

15 All correlations in this paper are computed as Pearson correlation coefficients, except where stated otherwise.

16 All statistical comparisons of correlation coefficients reported in this paper were conducted using the “cocor” package (Diedenhofen & Musch, 2015).

17 In conducting these analyses, we detected several apparent errors in the roughness values reported by Johnson-Laird et al. (2012). Here we use roughness values as computed by our new incon package.

18 https://www.jyu.fi/hytk/fi/laitokset/mutku/en/research/materials/mirtoolbox.

19 https://essentia.upf.edu.

20 https://github.com/pmcharrison/incon.

21 https://github.com/pmcharrison/inconData.

22 See https://github.com/pmcharrison/inconPaper for top-level source code.

23 As before, a *chord type* represents a chord as a set of intervals above an unspecified bass note.

24 Just tuning means expressing pitch intervals as small-integer frequency ratios. In Bowling et al. (2018), the eleven intervals in the octave were expressed as the following frequency ratios: 16:15, 9:8, 6:5, 5:4, 4:3, 7:5, 3:2, 8:5, 5:3, 9:5, 15:8, and 2:1.

25 In particular, we downloaded the “Exact 1-gram chord progressions” file from http://www.peachnote.com/datasets.html on July 2nd, 2018.

26 A frequency of *f* Hz corresponds to a MIDI note number of 69+12log_2_(*f*/440).
